# Gene expression signatures of response to fluoxetine treatment: systematic review and meta-analyses

**DOI:** 10.1038/s41380-025-03118-6

**Published:** 2025-07-17

**Authors:** David G. Cooper, J. Paige Cowden, Patrick M. Vo, Parker A. Stanley, Jack T. Karbowski, Victoria S. Gaertig, Caiden J. Lukan, Ariel D. Worthington, Caleb A. Class

**Affiliations:** Butler University, Department of Pharmaceutical Sciences, Indianapolis, IN USA

**Keywords:** Genetics, Neuroscience, Depression

## Abstract

**Background:**

Genomic (and other ‘omic) data have provided valuable insights on the pharmacological signatures of antidepressant response, but results from individual studies are largely heterogeneous. In this work, we synthesized gene expression data for fluoxetine treatment in both human patients and rodent models, to better understand biological pathways affected by treatment, as well as those that may distinguish clinical or behavioral response.

**Methods:**

Following the PRISMA guidelines, we searched the Gene Expression Omnibus (GEO) for studies profiling humans or rodent models with treatment of the antidepressant fluoxetine, excluding those not done in the context of depression or anxiety, in an irrelevant tissue type, or with fewer than three samples per group. Included studies were systematically reanalyzed by differential expression analysis and Gene Set Enrichment Analysis (GSEA). Individual pathway and gene statistics were synthesized across studies by three p-value combination methods, and then corrected for false discovery.

**Results:**

Of the 74 data sets that were screened, 20 were included: 18 in rodents, and two in tissue from human patients. Studies were highly heterogeneous in the comparisons of both treated vs. control samples and responders vs. non-responders, with 691 and 357 pathways, respectively, identified as significantly different between groups in at least one study. However, 18 pathways were identified as consistently different in responders vs. non-responders, including toll-like receptor (TLR) and other immune pathways. Signal transduction pathways were identified as consistently affected by fluoxetine treatment in depressed patients and rodent models.

**Discussion:**

These meta-analyses confirm known pathways and provide new hints toward antidepressant resistance, but more work is needed. Most included studies involved rodent models, and both patient studies had small cohorts. Additional large-cohort studies applying additional ‘omics technologies are necessary to understand the intricacies and heterogeneity of antidepressant response.

## Introduction

Major depressive disorder (MDD) is a top cause of disability worldwide, and antidepressant medications remain a common first-line therapy [1]. Although almost all share a similar presumed mechanism of action modulating levels of neurotransmitters such as serotonin and norepinephrine, their efficacy and adverse effects can vary widely—approximately half of patients do not respond to their first prescribed antidepressants, and the challenge in selecting the optimal treatment stems in part from our limited understanding of their specific mechanisms [2, 3]. Extensive research has sought to unravel the complex processes involved in antidepressant treatment in both the central and peripheral nervous systems [4–6]. The use of computational chemistry and “omics” methods have helped in this and other areas, providing leads for new drugs, insights into their mechanisms of action, and potential signatures of response [7–9]. However, these methods have not been the panaceas that some may have hoped, as heterogeneity between patients, interactions between multiple levels of biology, and environmental and other factors can be difficult to capture [3]. So while progress has been made to understand the mechanisms of action of antidepressants, many of the nuances remain enigmatic.

The use of systematic reviews in mental disorders and other fields has dramatically increased in recent years to synthesize the wealth of clinical data being generated [10–13]. However, these guidelines have been applied less frequently in the use of gene expression data, including one non-PRISMA systematic review and meta-analysis of gene expression signatures corresponding to fluoxetine treatment in rodents [14–16]. These studies generally focus on a specific tissue type in either humans or rodents, and translating between organisms remains difficult despite recent advances. Although over 15,000 annotated human genes have orthologs in mice, their function is not always shared, which has pharmacological implications; in cancer for example, fewer than 8% of drugs successfully translate from animal models to clinical trials [17–19]. Niculescu et al. have employed convergent functional genomics to identify consistent genes in independent studies of patients and rodent models for risk prediction in depression, schizophrenia, and other psychiatric disorders to find biomarkers of clinical value [20–22]. Alternatively, meta-analyses considering biological pathways do not find specific biomarkers but allow us to focus on the shared biological effects between studies, which may be more consistent across studies when a phenotype arises due to subtle but concerted changes of multiple genes within pathways [23–25]. In this systematic review and meta-analysis, we will apply both the pathway- and gene-level approach to summarize and potentially identify new biological pathways of antidepressant treatment and response.

The objectives of this systematic review are to synthesize the evidence for gene expression modification by treatment with the SSRI fluoxetine, and whether gene expression levels distinguish clinical or behavioral response to treatment, across multiple tissue types in humans and rodent models. In this paper, we use “Response Signatures” to refer to gene expression distinguishing those with good vs. poor response to fluoxetine, while “Treatment Signatures” signifies differences between fluoxetine treatment vs. control. We select fluoxetine for this review due to the abundance, yet also heterogeneity, in gene expression studies already conducted [15, 26]. To our knowledge, this is the first systematic meta-analysis of gene expression data to investigate behavioral or clinical response to an antidepressant, as well as the first to integrate biological pathways across multiple organisms and tissue types; in addition to synthesizing studies profiling gene expression across varying brain regions, we include studies in peripheral tissue to identify shared pathways or potential biomarkers that would be easier to assess in clinical settings. We apply a consistent analysis pipeline across all studies, so that we do not rely on varying definitions of statistical significance applied by different researchers. Results of the meta-analyses may be applied to improving the prediction and assessment of fluoxetine response shortly after treatment, and they may provide hints for combination therapies or new drug development for MDD.

## Methods

The Gene Expression Omnibus (GEO) was identified as the main database for this systematic review, due to its primary focus on gene expression data and the relative consistency of data deposits and formatting [27]. PubMed was identified as a contingent database if the GEO search resulted in fewer than five studies that passed screening (it was not used since greater than five GEO studies passed screening). As one of the most prescribed and studied antidepressants, fluoxetine was selected as the focus of this systematic review; we were more inclusive regarding organisms and tissue type, allowing us to identify both heterogeneity and potential consistencies. The GEO search was conducted on 4 April 2023, using the following keywords: *(fluoxetine) OR (selective serotonin reuptake inhibitor) OR (ssri)*. Results were filtered within GEO using *Entry type* = *Series* (to return full data sets rather than individual samples), *Organism* = *homo sapiens, mus musculus*, or *rattus norvegicus*, and *Study type* = *Expression profiling by array* or *expression profiling by high throughput sequencing*. The resulting data series were manually filtered by authors DC and CC based on the exclusion criteria: studies were excluded if they were not primarily focused on depression or anxiety, were not conducted in a relevant tissue type or genetic background (cancer cell lines, for example), did not involve fluoxetine treatment, or if there were fewer than three samples per group. Decisions were made based on review of the GEO series abstract and sample metadata. If unclear, any cited publication and its Supplementary Materials were consulted for additional information.

Data extraction, transformation, synthesis, and assessment were conducted using R version 4.2.3 [28]. Each individual analysis was completed by one author and checked by DC or CC. All analysis code and results are available for download (see **Code Availability**). Gene expression data were downloaded using the R library GEOquery, and then checked for completeness [29]. Data sets that were not retrieved properly using GEOquery were downloaded directly from the GEO website and imported into R for analysis. Basic quality control was assessed by visualizing and comparing gene expression distributions, principal components analysis, and between-sample correlations. Clear outlier samples (greater than six standard deviations from the mean of all samples in the study based on the first two principal components) were removed prior to any subsequent analyses. Gene-level differential expression analysis was conducted using the R libraries DESeq2 for RNA sequencing data, or limma for microarray data [30, 31]. All differential expression analyses were conducted as *group* vs. *group*, with the main variables being treatment status or response (as appropriate). Other variables sought were tissue type, time point, and participant/rodent ID. For each comparison, the effect measures used in subsequent steps were the log2 fold change (log2FC), nominal p-value, and t-statistic. When multiple tissue types were present in a study, separate analyses were conducted within each tissue. Risk of bias was assessed by evaluating the provided characteristics of the study populations.

Differential expression results for each comparison were summarized to the pathway level using the fgsea R library for Gene Set Enrichment Analysis (GSEA), using the Reactome and KEGG pathway gene sets accessed through the Consensus Pathway Database (CPDB) [32–36]. The CPDB provides orthologous gene sets for human and mouse genomes, allowing for the synthesis of human and rodent models at the pathway level (for rat data sets, gene IDs were converted to mouse IDs by homology prior to GSEA). A ranked list of differential expression t-statistics from each comparison was input to fgsea, and relevant effect measures for each pathway were the normalized enrichment score (NES) providing the magnitude of gene expression enrichment in one group or the other, and nominal p-value.

**Fig. 1:**
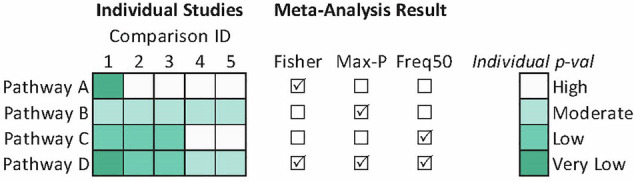
Demonstration of meta-analysis methods used in this study. Meta-analyses were used to synthesize pathway and differential expression analyses. Heatmap on left shows GSEA nominal p-values for four example pathways in five comparisons. Checkboxes indicate which meta-analysis method(s) would identify that pathway as significantly enriched.

Results across comparisons were synthesized using MetaDE [37]. For synthesis, we grouped studies based on the specific comparisons they made: (1) good vs. poor response, (2) fluoxetine treatment vs. control, (3) fluoxetine treatment vs. control among depressed patients and stressed rodent models, and (4) fluoxetine treatment vs. control among unstressed rodents. Studies were synthesized across organisms, and across studies profiling the same organism. P-value combination methods were selected due to substantial heterogeneity between studies (organism, tissue type, and gene expression platform). Within organisms, p-values were synthesized at both the gene and pathway level, while across-organism syntheses were only conducted at the pathway level. Fisher’s method was used to identify genes or pathways that were differentially expressed in *any study*, while Wilkinson’s method (*Max-P*) was used to identify genes or pathways that were consistently differentially expressed *across studies* [38–40]. The meta-analysis p-values were corrected for false discovery using the method of Benjamini and Hochberg, resulting in q-values [41]. As an intermediate method, we also selected genes or pathways with nominal *p* < 0.05 in greater than half of synthesized comparisons (Frequency of 50%, or *Freq50*). A depiction of these methods is provided in Fig. 1. Synthesis results were displayed using Venn diagrams to show how many genes/pathways were identified by each meta-analysis method, and bar graphs showing pathways identified by Max-P with q < 0.05.

Heterogeneity among study results was investigated using subgroup analysis within organisms or tissue types, as well as heatmaps of pathway NESs across studies. To estimate certainty of the meta-analyses, consistency of the direction of enrichment was estimated by simple vote-counting across comparisons: a pathway or gene was assigned +1 for each comparison where it was significantly enriched in responders with nominal *p* < 0.05, or −1 if enriched in non-responders. These were summed across all comparisons, and numbers further from zero indicate greater consistency in results that are statistically significant within comparisons. For genes or pathways identified as consistently differentially expressed across studies, we generally expect them to agree in the direction of the effect (i.e. overexpressed in responders or non-responders). This is not assured, as differing and even opposite effects have been reported in some cases [42, 43].

Sensitivity analyses were performed to assess the sensitivity of the meta-analyses results to the specific datasets included, as well as to the type of pathway analysis method employed (Supplementary Methods). Additionally, we compared our results with those from two larger-cohort patient studies conducted in different contexts: one comparing patients treated with duloxetine or placebo, and a naturalistic study comparing patients with remitted or current MDD [44, 45].

## Results

**Fig. 2:**
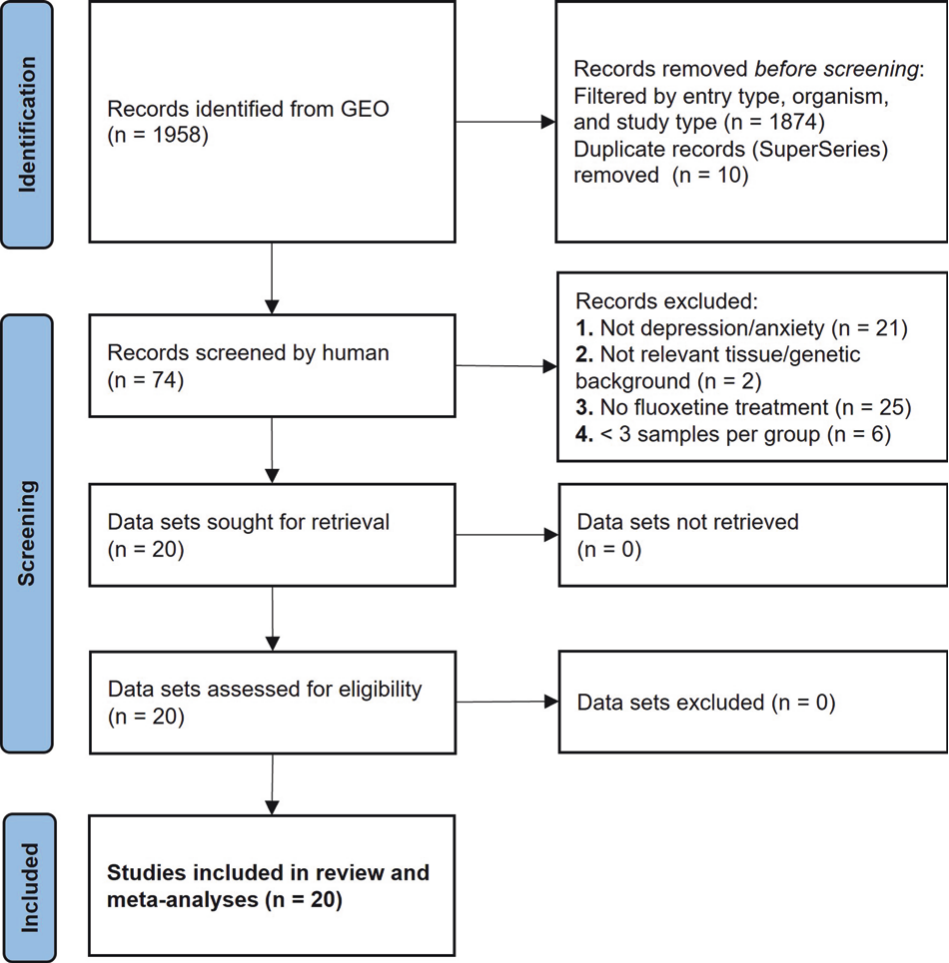
PRISMA flow diagram. Of the 1958 records returned from GEO, 74 were manually screened and 20 were included in our review and meta-analyses.

The initial keyword search resulted in 1958 entries, which was filtered to 84 data sets (known in GEO as “series”) using automatic filtering for Entry Type, Study Type, and Organism (Fig. 2). Most of the automatically excluded entries were individual samples, which were not targets for this study and generally were included in one of the returned data sets. 10 “SuperSeries” were removed as duplicates, as they contained one or more individual series returned by the search. 74 data series were manually assessed for the exclusion criteria (Supplementary Table 1), resulting in 20 selected for inclusion [46–61] (Table 1, Fig. 2). Of the 20 included data sets, two profiled tissue from patients diagnosed with MDD: one profiled gene expression in whole blood from adolescent females before and after eight weeks of continuous fluoxetine treatment [58], while the other profiled lymphoblastoid cell lines (LCLs) developed from 10 patients with known antidepressant response based on change in score by Hamilton Depression Rating Scale (HDRS). These LCLs were treated with fluoxetine or control in vitro for three weeks [48].

**Table 1:** 20 data sets included in synthesis.

ID	Comparisons	GEO Series Accession Number	Citation	Platform	Organism	Tissue(s)	Samples	Stress Method / Diagnosis	Fluoxetine Treatment	Drug Response Determination
DS1	(a–d) Cortex	GSE28644	Benton et al. [46]	Microarray	*Mus musculus*	Cortex	60 (30/group)	N/A	3-weeks 18 mg/kg/day	Open-field test
	(a) Response									
	(b) Treatment									
	(c) Treatment (Non-responders)									
	(d) Treatment (Responders)									
DS2	(a, c, e, g) Dorsal Dentate Gyrus	GSE43261	Samuels et al. [47]	Microarray	*Mus musculus*	Dorsal dentate gyrus, Ventral dentate gyrus	38 (4 or 8/group)	Corticosterone	>1-week 160 mg/L in drinking water	Novelty suppressed feeding, Forced swim test
	(b, d, f, h) Ventral Dentate Gyrus									
	(a, b) Reponse									
	(c, d) Treatment									
	(e, f) Treatment (Non-responders)									
	(g, h) Treatment (Responders)									
DS3	(a–d) Lymphoblastoid Cell Lines	GSE83386	Breitfeld et al. [48]	Microarray	*Homo sapiens*	Lymphoblastoid cell lines	20 (10/group)	Major Depressive Disorder	3-weeks 0.5 µg/mL	Hamilton Depression Rating Scale
	(a) Response									
	(b) Treatment									
	(c) Treatment (Non-responders)									
	(d) Treatment (Responders)									
DS4	(a, c, e, g, i) Anterior Cingulate Cortex	GSE84183	Hervé et al. [49]	Microarray	*Mus musculus*	Anterior cingulate cortex, Dentate gyrus	64 (8/group)	7-weeks unpredictable chronic mild stress	5-weeks 120 mg/L in drinking water	Coat state measurement
	(b, d, f, h, j) Dentate Gyrus									
	(a, b) Reponse									
	(c, d) Treatment (Stressed)									
	(e, f) Treatment (Unstressed)									
	(g, h) Treatment (Non-responders)									
	(I, j) Treatment (Responders)									
DS5	(a–e) Blood	GSE84184	Hervé et al. [49]	Microarray	*Mus musculus*	Whole blood	32 (8/group)	7-weeks unpredictable chronic mild stress	5-weeks 120 mg/L in drinking water	Coat state measurement
	(a) Reponse									
	(b) Treatment (Stressed)									
	(c) Treatment (Unstressed)									
	(d) Treatment (Non-responders)									
	(e) Treatment (Responders)									
DS6	(a, c, e, g) Whole Cortex	GSE202172	Sargin et al. [50]	RNA-Seq	*Mus musculus*	Whole cortex, S100a10 cortical cells	32 (4/group)	7-weeks single-housing	3-weeks 167 mg/L in drinking water	Homecage time spent in shelter zone
	(b, d, f, h) S100a10 TRAP									
	(a, b) Reponse									
	(c, d) Treatment									
	(e, f) Treatment (Non-responders)									
	(g, h) Treatment (Responders)									
DS7	(-) S100a10 Cells	GSE35761	Schmidt et al. [51]	Microarray	*Mus musculus*	S100a10 cortical cells	6 (3/group)	N/A	15–18 days 167 mg/L in drinking water	None
	(-) Treatment									
DS8	(-) Glt25d2 Cells	GSE35763	Schmidt et al. [51]	Microarray	*Mus musculus*	Glt25d2 cortical cells	6 (3/group)	N/A	15–18 days 167 mg/L in drinking water	None
	(-) Treatment									
DS9	(-) Hippocampus	GSE42940	Sarkar et al. [52]	Microarray	*Rattus norvegicus*	Hippocampus	8 (4/group)	N/A	3-weeks 10 mg/kg/day	None
	(-) Treatment									
DS10	(a, d) Striatum	GSE48951	Korostynski et al. [53]	Microarray	*Mus musculus*	Striatum	60 (12/group)	N/A	1-dose 20 mg/kg	None
	(a) Treatment 1 h									
	(b) Treatment 2 h									
	(c) Treatment 4 h									
	(d) Treatment 8 h									
DS11	(-) Dentate Gyrus	GSE56028	Patrício et al. [54]	Microarray	*Rattus norvegicus*	Dentate gyrus	21 (3/group)	6-weeks unpredictable chronic mild stress	2-weeks 10 mg/kg/day	None
	(-) Treatment									
DS12	(-) Brain	GSE86392	Wang et al. [55]	RNA-Seq	*Rattus norvegicus*	Hippocampus, Frontal cortex, Pituitary gland	12 (3/group: 1/tissue)	4-weeks of chronic restraint stress	4-weeks 10 mg/kg/day	None
	(-) Treatment									
DS13	(-) Glioma	GSE89873	Malik et al. [56]	Microarray	*Rattus norvegicus*	C6 glioma cells (model of astrocytes)	28 (4 or 6/group)	N/A	2 h 25 µM	None
	(-) Treatment									
DS14	(-) Hippocampus	GSE109445	N/A	RNA-Seq	*Rattus norvegicus*	Hippocampus	12 (3/group)	5-weeks chronic unpredictable stress	1-week 10 mg/kg/day	None
	(-) Treatment									
DS15	(-) Prefrontal Cortex	GSE118668	Hagihara et al. [57]	Microarray	*Mus musculus*	Prefrontal cortex	16 (8/group)	N/A	3-weeks 15 mg/kg/day	None
	(-) Treatment									
DS16	(-) Dentate Gyrus	GSE118669	Hagihara et al. [57]	Microarray	*Mus musculus*	Dentate gyrus	16 (8/group)	N/A	3-weeks 15 mg/kg/day	None
	(-) Treatment									
DS17	(-) Blood	GSE128387	Torres et al. [58]	Microarray	*Homo sapiens*	Whole blood	32 (15–17/group)	Major Depressive Disorder	8-weeks, dosage not specified	None
	(-) Treatment									
DS18	(-) Amygdala	GSE150431	Rajkumar et al. [59]	RNA-Seq	*Mus musculus*	Amygdala	48 (8/group)	N/A	2 days, 5 mg/kg/day	None
	(-) Treatment									
DS19	(a-aa) 27 Brain Regions	GSE194289	Rayan et al. [60]	RNA-Seq	*Rattus norvegicus*	27 brain regions	212 (~4/group)	N/A	6-weeks 18 mg/kg/day	None
	*See* Supplementary Table 2									
	(a-aa) Treatment									
DS20	(-) Hippocampus	GSE205325	Demin et al. [61]	RNA-Seq	*Rattus norvegicus*	Hippocampus	21 (3/group)	12-weeks chronic unpredictable stress	4-weeks 5 mg/kg/day	None
	(-) Treatment									

“Comparisons” were either Responders vs. Non-responders (“Response”) or Fluoxetine Treated vs. Untreated (“Treatment”). Letters in parentheses in Comparisons column are used as reference in subsequent figures.

The remaining 18 studies profiled gene expression with fluoxetine treatment in rodent models (11 in mice, seven in rats). Stress models were applied in nine of these studies to achieve anxious and/or depressive behavior: five employed chronic mild stress [49, 54, 61–63], while the others used either chronic restraint stress [55], single housing [50], injection with corticosterone [47], or selective breeding for high anxiety [56]. Five studies included a classification of response in the treated animals, which was assessed using a behavioral method such as the open-field test (OFT) for measurement of anxiety [46] or the forced swim test (FST) for despair [47]. One study did not induce stress but did measure behavioral response: anxiety was measured by OFT after three weeks of fluoxetine or vehicle treatment, and response was defined based on the ratio of scores between the fluoxetine and vehicle groups [46]. One mouse study profiled whole blood, but the majority collected samples from one or more brain regions for gene expression profiling: the most common was hippocampal tissue (particularly dentate gyrus, included in five studies [47, 49, 54, 57, 60]). Two studies applied translating ribosome affinity purification (TRAP) with a focus on S100a10 neurons, which have been shown to play a critical role in mediating antidepressant response through p11 expression; one study compared these cells to those from Glt25d2 cells, a layer 5 cortical cell that does not express p11, while the other includes whole cortex profiling in addition to S100a10 [50, 51]. One profiled fluoxetine effects across 27 brain regions in rats, identifying region-specific differential expression signatures in both bulk and single-cell RNA-Seq data (we did not include single-cell data in this work) (Supplementary Table 2) [60].

As is common with rodent models, males were utilized in 17 of the 18 studies, demonstrating a substantial risk of bias. One study profiled gene expression with fluoxetine and imipramine treatment in female mice, which had been shown to exhibit behavioral despair by FST and tail suspension test (TST) after reduction in *Brd1* expression (this was not observed in male mice with reduced *Brd1* expression) [59]. Conversely, the two human studies were biased toward female inclusion, with one including adolescent females only [58], and the other including eight females and two males in the microarray analysis [48]. For the six data sets synthesized for Response Signatures, all five mouse data sets included males, while the patient cohort was biased toward females.

### Response signatures

**Fig. 3:**
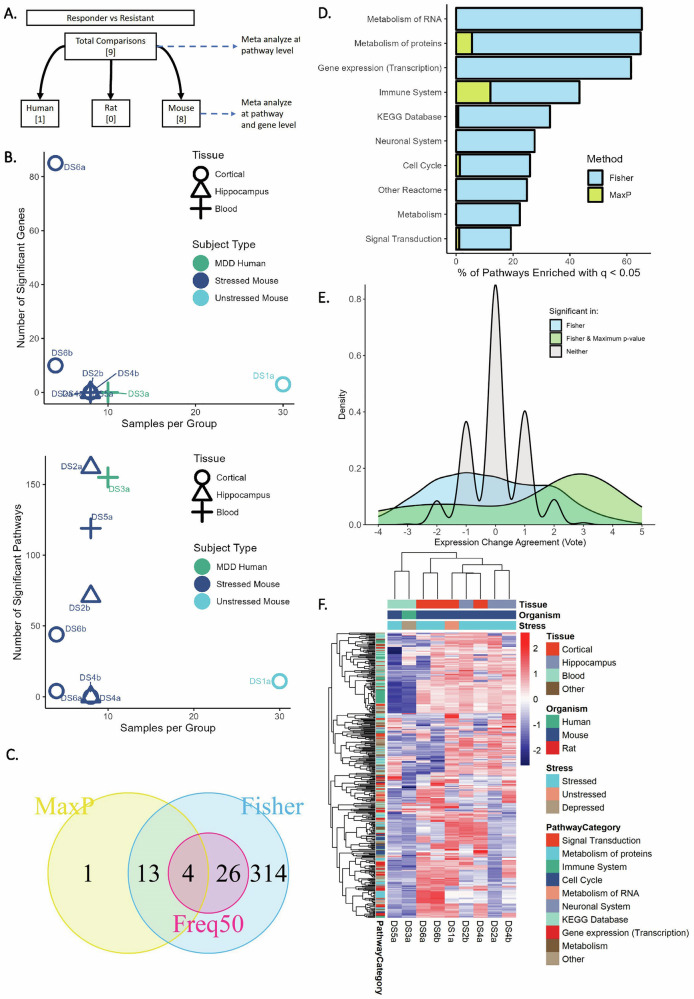
Synthesis of studies for behavioral or clinical response. **A** Individual comparisons used in meta-analysis. **B** Number of genes differentially expressed (top) and pathways enriched (bottom), vs. number of samples per group in each of the nine comparisons of responders vs. non-responders (q < 0.05). **C** Pathways identified as significantly enriched (q < 0.05) across the nine comparisons by the three meta-analysis methods. **D** Percentage of pathways significantly enriched in responders or non-responders from each of the Reactome categories (“Top Level”) or KEGG Database. x-axis indicates percentage of pathways from a certain category identified as enriched. **E** Density plot showing distribution of Vote Sums across pathways, colored by meta-analysis result. Positive scores indicate enrichment in responders, negative in non-responders. One pathway was identified as enriched by Max-P but not Fisher’s method, with Vote total +1 (Cell death signaling, *not shown*). **F** Heatmap of Normalized Enrichment Score (NES) across comparisons for pathways identified as enriched in any comparison (Fisher’s q < 0.05). Positive NES indicates enrichment in responders.

Meta-analysis by Fisher’s method, the Max-P method, and the Freq50 method (Fig. 1) were applied on nine comparisons from six data series to synthesize gene expression signatures associated with clinical or behavioral response to fluoxetine (Fig. 3A). Systematic re-analysis of individual comparisons resulted in widely varying numbers of differentially expressed genes and pathways after false discovery correction (Fig. 3B). DS6a had the greatest number of significant genes at 85, while four comparisons showed no genes differentially expressed with q < 0.05. Conversely, statistically significant pathway enrichment was observed for all studies except DS4. In particular, the two studies profiling blood tissue (one in MDD patients, one in stressed mice) and two comparisons profiling dentate gyrus in stressed mice showed the greatest number of enriched pathways, suggesting slight gene expression changes that were coordinated across multiple pathways. Nominal p-values were synthesized across all comparisons for each Reactome and KEGG pathway and then corrected for false discovery to result in q-values. After false discovery correction, meta-analysis identified 357 pathways (over 30% of the pathways analyzed) enriched in at least one comparison (q < 0.05 by Fisher’s method), 18 pathways enriched across comparisons (Max-P), and 30 pathways enriched in at least five comparisons (Freq50) (Fig. 3C). Pathways involving metabolism of proteins or RNA, transcription, or the immune system were most likely to be enriched (Fig. 3D).

We then considered direction of effect through vote-counting across comparisons: a pathway was assigned +1 if significantly enriched in responders, or −1 if enriched in non-responders. The distribution of vote sums for all pathways is provided in Fig. 3E. Pathways that were not identified as enriched by either meta-analysis method had distributions centered around zero, with 92% of pathways ranging from −1 to +1, indicating the expected low agreement. Those identified as significant by meta-analysis had a wider range, indicating more consistent enrichment in responders or non-responders. However, the greatest vote sum magnitude was +5 (of a possible 9 if all comparisons were significant in the same direction), indicating moderate certainty of results.

The Normalized Enrichment Scores (NES) by GSEA for pathways enriched by Fisher’s method are presented in Fig. 3F. Strong heterogeneity is observed across comparisons, and individual comparisons share relatively few enriched pathways between them as quantified through Jaccard indices (Supplementary Fig. 1). Some patterns can be detected: the two studies profiling expression in mouse blood or human lymphoid-derived cells cluster together (sharing 99 affected pathways, Jaccard index = 0.27), while the seven from brain tissue comprise the other cluster. A group of immune pathways show strong fluoxetine-induced upregulation in resistant blood samples, but weak, opposite enrichment across brain samples from responding mice. Other clusters show heterogeneous patterns within the set of comparisons profiling brain tissue, and none are enriched in the same direction across all comparisons, as evidenced by the vote sums.

**Fig. 4:**
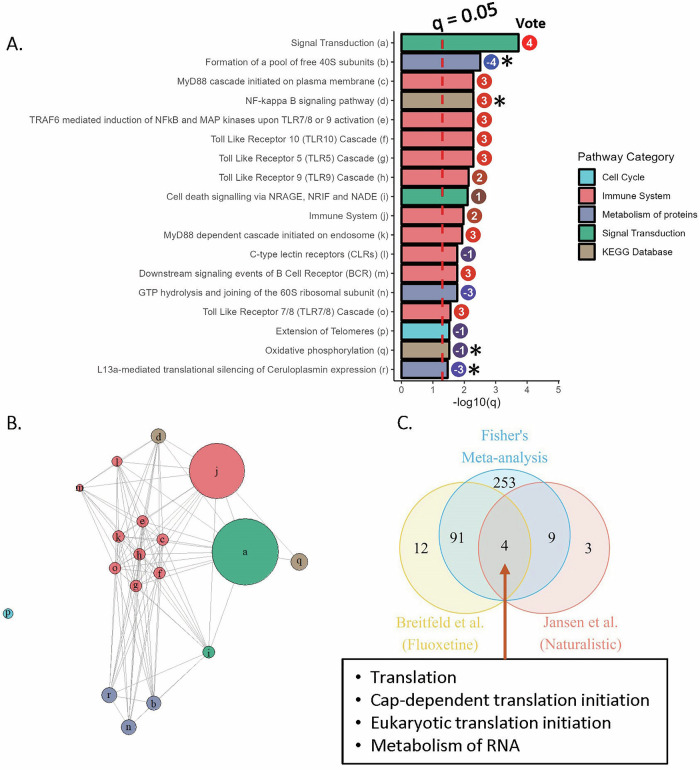
Pathways consistently altered between responders and non-responders. **A** q-value by Max-P and Vote Sums for pathways identified as enriched across the nine comparisons of responders vs. non-responders (q < 0.05 by Max-P). * indicates pathways also enriched by Freq50 (*p* < 0.05 in greater than half of comparisons). **B** Network diagram showing similarity between pathways, with labels provided in **A**. Pathways sharing more genes are plotted closer together. **C** Number of pathways identified as significantly enriched in responders or non-responders (q < 0.05) by Fisher’s meta-analysis, DS3 (Human LCL’s), and the naturalistic study by Jansen et al.

Meta-analysis statistics for pathways identified as consistently enriched by Max-P (q < 0.05) are presented in Fig. 4A, and a network diagram showing similarity between these gene sets is presented in Fig. 4B (overlap between gene sets can be seen in Supplementary Fig. 2). Signal Transduction (a top-level Reactome pathway with over 2000 genes) was the most consistently enriched pathway with q < 0.001 by Max-P and a vote sum of +4, indicating somewhat consistent fluoxetine-induced upregulation in good responders. 10 immune pathways were identified as consistently enriched, including five toll-like receptor (TLR) pathways and two MyD88 cascade pathways; these seven pathways are highly overlapping, sharing over 95% of genes. Additionally, the NF-kappa B signaling pathway, C-type lectin receptors (CLRs), Downstream signaling events of B Cell Receptor (BCR), and the top-level Immune System pathway were identified by meta-analysis. These were slightly upregulated in good responders by total vote, except for the CLRs pathway (Vote = −1, upregulated in non-responders in patient-derived LCL’s).

Pathways related to protein metabolism were most consistently upregulated in poor responders, including the substantially overlapping pathways related to 40S and 60S ribosomal subunits (vote sums of −4 and −3, respectively) and L13a-mediated translational silencing of Ceruloplasmin expression (−3). These were enriched in non-responders in both comparisons involving blood-derived tissue, as well as some comparisons in brain tissue. Additionally, Extension of Telomeres (q = 0.03, with negligible overlap with the other enriched pathways in Fig. 4B) was enriched by Max-P with a vote sum of −1.

We also compared our meta-analysis results to two larger-cohort studies conducted in different contexts: Belzeaux et al. profiled gene expression from 424 patients treated with the SNRI duloxetine or placebo, and Jansen et al. compared gene expression from patients with remitted (*N* = 635) or current MDD (*N* = 882), many of whom (but not all) took some antidepressant [44, 45]. We identified no enriched pathways with q < 0.05 from the former, and 16 enriched pathways from the latter study: these pathways did not overlap with those identified via Max-P for fluoxetine response, although 13 overlapped with those identified via Fisher’s meta-analysis (Fig. 4C). 4 translation-related pathways also overlapped with the results of DS3 profiling patient LCL’s, but they did not agree in the direction of enrichment. One immune pathway (BCR signaling) was identified as upregulated in remitted MDD in the Jansen et al. study and by Fisher’s meta-analysis, while Downstream signaling events of BCR was identified as upregulated in good responders by Max-P meta-analysis.

Subgroup and sensitivity analyses are described in the Supplementary Results. Briefly, the pathway results were only slightly sensitive to the inclusion of gene expression profiling in blood samples, as all pathways identified as consistently affected in the brain were also affected in blood; however, they were sometimes affected in the opposite direction. We also compared results from two studies that allowed us to compare responders and non-responders naïve to fluoxetine treatment, identifying 13 pathways enriched in both comparisons.

### Treatment signatures

**Fig. 5:**
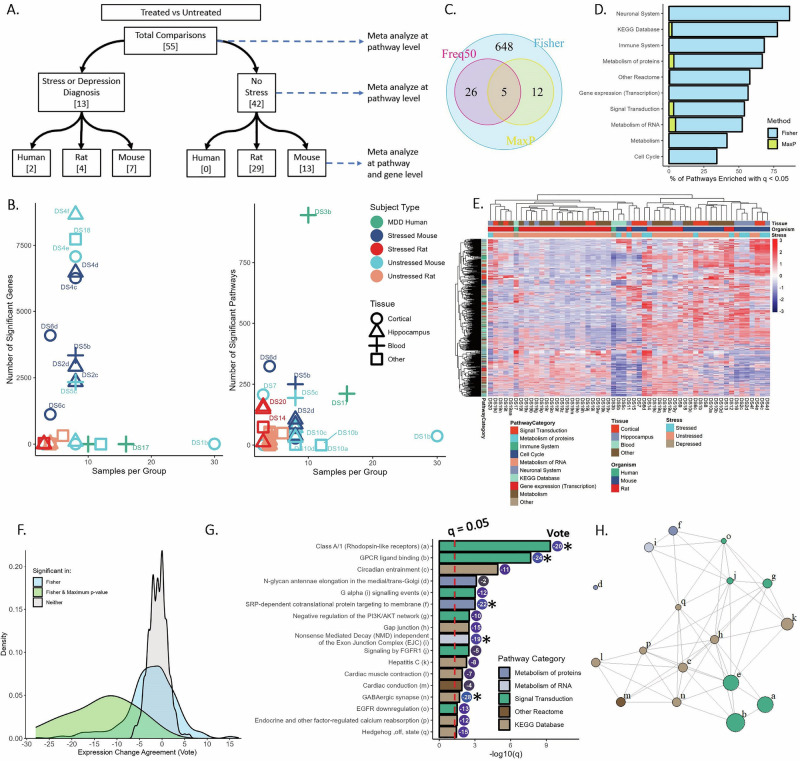
Synthesis of studies for fluoxetine treatment effects. **A** Individual comparisons used in meta-analysis. **B** Number of genes differentially expressed (left) and pathways enriched (right), vs. number of samples per group in each of the 55 comparisons of fluoxetine treated vs. control samples (q < 0.05). **C** Pathways identified as significantly enriched (q < 0.05) across the 55 comparisons by the three meta-analysis methods. **D** Percentage of pathways significantly affected by fluoxetine treatment from each of the Reactome categories (“Top Level”) or KEGG Database. **E** Heatmap of Normalized Enrichment Score (NES) across comparisons for pathways identified as enriched in any comparison (Fisher’s q < 0.05). Positive NES indicates increased expression with fluoxetine treatment. **F** Density plot showing distribution of Vote Sums across pathways, colored by meta-analysis result. **G** q-value by Max-P, and Vote Sums for pathways identified as significant across the 55 comparisons of treated vs. untreated (q < 0.05 by Max-P). * indicates pathways also enriched by Freq50 (*p* < 0.05 in greater than half of comparisons). **H** Network diagram showing similarity between pathways, with labels provided in **G**. Pathways sharing more genes are plotted closer together.

The same meta-analyses were applied on 55 comparisons of treated vs. control samples from 20 data sets, to synthesize gene expression changes due to treatment (Fig. 5A). First, each data set was systematically reanalyzed, again providing widely varying results (Fig. 5B): after false discovery correction, the number of differentially expressed genes ranged from zero (in 19 comparisons) to 8663, and the number of significantly enriched pathways ranged from zero (in 6 comparisons) to 945. Again, studies profiling gene expression in blood generally identified fewer differentially expressed genes, but they identified greater than 200 enriched pathways spanning many biological processes. Additionally, studies profiling fluoxetine treatment in unstressed rodents identified few enriched pathways, with DS5c (whole blood) and DS7 (S100a10 cortical cells) as notable exceptions. Fisher’s meta-analysis identified 691 pathways as enriched in at least one comparison; of these, 31 were enriched with nominal *p* < 0.05 in more than half of the comparisons, and 17 were identified as consistently enriched by the Max-P method (Fig. 5C).

The pathways identified as enriched in any of the 55 comparisons (61% of all pathways analyzed) are represented by all pathway categories, but most frequently components of Neuronal System, KEGG database, and immune system (Fig. 5D). Heterogeneous enrichment patterns are again observed across studies (Fig. 5E, Supplementary Fig. 6, Supplementary Fig. 7). Some clustering was observed by organism and application or absence of a stress model. Tissue type again played a role, as three of the four comparisons from blood clustered tightly together, and many comparisons from hippocampus and cortex clustered together. Comparisons from the same study frequently clustered together as well; comparisons from DS2 profiling dorsal and ventral dentate gyrus (Jaccard = 0.56) and DS5 profiling stressed and unstressed mice (Jaccard = 0.59) showed the greatest overlap in enriched pathways.

Overall, pathways identified only by Fisher’s method have a wider distribution of vote sums (but still highly concentrated between +6 and −6, of a possible 55 for perfect agreement) than those that are not significantly enriched, while those identified by Max-P are skewed toward the left indicating downregulation by treatment (Fig. 5F). The 17 pathways enriched by Max-P (Fig. 5G) mainly come from the Signal Transduction category of Reactome or the KEGG Database. Two G protein-coupled receptor (GPCR) binding pathways were most consistently downregulated by fluoxetine according to Max-P meta-analysis, and GABAergic synapse is most consistently decreased by treatment according to vote (−28, q = 0.006). Aside from GPCR pathways, there was not substantial overlap between genes within most enriched pathways (Fig. 5H and Supplementary Fig. 8).

**Fig. 6:**
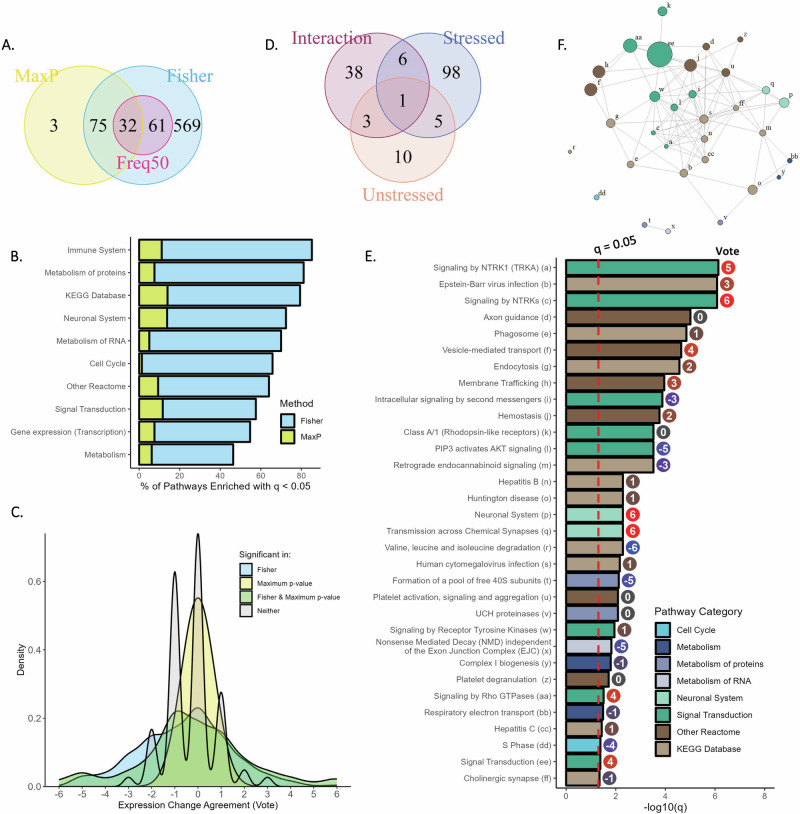
Subset analysis for fluoxetine treatment effects. **A** Pathways identified as significantly enriched (q < 0.05) across the 13 comparisons of treatment effects in MDD patients or stressed rodent models. **B** Percentage of pathways significantly affected by fluoxetine treatment in MDD patients or stressed rodent models from each of the Reactome categories (“Top Level”) or KEGG Database. **C** Density plot showing distribution of Vote Sums across pathways, colored by meta-analysis result. Positive scores indicate increased expression with fluoxetine. **D** Overlap of pathways identified as statistically significant by Max-P meta-analysis of treated vs. untreated samples in stressed rodents or depressed patients, in unstressed rodents, and pathways identified by interaction analysis comparing fluoxetine effects in stressed vs. unstressed mice. **E** q-value by Max-P and Vote Sums for pathways identified as significant across the 13 comparisons in MDD patients or stressed rodents (q < 0.05 by Max-P), subset to include only pathways also enriched by Freq50 (*p* < 0.05 in greater than half of comparisons). **F** Network diagram showing similarity between pathways, with labels provided in **E**.

Subset meta-analysis was conducted for 13 comparisons from 10 data sets profiling fluoxetine treatment of stressed rodents and human MDD patients, as these studies may be more relevant in understanding fluoxetine’s mechanism of action in the context of MDD. 737 pathways were identified as enriched in at least one comparison, and 110 were identified as consistently enriched by Max-P (Fig. 6A). Greater enrichment is observed in immune pathways in this subset analysis as compared with the full analysis (Fig. 6B). Generally low agreement between studies is observed, with vote sums ranging from −2 to +2 for 98.7% of pathways not identified as enriched by meta-analysis (Fig. 6C). 24% of pathways identified by both Fisher’s and the Max-P method had vote sums outside this range. Additionally, we conducted interaction analysis to identify genes differentially affected by fluoxetine treatment in one study that profiled both stressed and unstressed mice: 48 pathways were identified in this analysis, with 9 overlapping with the subset meta-analysis in stressed or unstressed mice (Fig. 6D and Supplementary Fig. 9).

The 32 pathways identified by both Max-P and Freq50 are presented in Fig. 6E (all 110 pathways identified by Max-P are presented in Supplementary Fig. 10). Most significantly enriched are two NTRK signaling pathways, both with q < 0.001 and vote sums of +5 and +6 respectively (of 13 possible), indicating moderate certainty, along with Epstein-Barr virus infection (q < 0.001, +3). A network graph showing overlap between enriched pathways indicates groups of connected pathways from the KEGG Database and Reactome Signal Transduction, in addition to other smaller groups (Fig. 6F). We compared the meta-analysis results with the larger-cohort study by Belzeaux et al. profiling gene expression from whole blood in MDD patients before and after duloxetine treatment, identifying 21 enriched pathways (q < 0.05) in common with the Max-P meta-analysis results, largely related to signal transduction and RNA/protein metabolism. Direction of enrichment agreed between this study and the meta-analysis (by Vote) for 13 of 18 pathways (the other three identified by Max-P had Vote sums of zero) (Supplementary Fig. 11).

Subgroup and sensitivity analyses are described in the Supplementary Results. Briefly, the greatest number of consistently differentially expressed genes (Max-P) was identified in stressed mice, with Brain-derived neurotrophic factor (*Bdnf*) identified as most consistent (q«0.001 by Max-P, upregulated with treatment in all 7 comparisons). We also conducted meta-analysis of treatment effects within responding and non-responding subgroups, identifying substantially more consistently affected pathways in responders (Supplementary Fig. 14). Finally, analysis with DAVID identified zero gene sets as consistently differentially expressed across all comparisons in stressed rodents and depressed humans, although 42 were identified by Freq50 and reasonable consistency was identified comparing Fisher’s meta-analysis statistics of KEGG pathways returned by GSEA and DAVID (Supplementary Fig. 16).

## Discussion

Across nine independent comparisons of fluoxetine responders vs. non-responders from six gene expression data sets, 18 pathways were identified as consistently enriched by Max-P meta-analysis of GSEA results. This was surprising, as these studies profiled different tissue types and employed varying stress models in mice--in addition to one which did not induce stress, and another using samples from MDD patients. This also demonstrated the ability of pathway methods vs. individual gene meta-analysis in identifying consistency: among the eight comparisons in mice, zero genes were identified with consistent differences between responders and non-responders by Max-P, and only four were identified as differentially expressed in greater than half of comparisons. Less surprisingly, over three hundred pathways were identified as enriched in at least one comparison by Fisher’s method, demonstrating the expected heterogeneity in behavioral response markers between studies. Some of this heterogeneity can be explained by tissue type, as comparisons from similar tissues clustered nearest each other (Fig. 3F). Thus, we also conducted separate meta-analysis excluding studies profiling expression in blood, but we saw only a slight effect on the meta-analysis result by Max-P, indicating that pathways enriched in brain tissue were generally enriched in blood as well, but sometimes in the opposite direction.

Immune pathways were well represented among the 18 pathways that were consistently different between responders and non-responders across organisms and tissue types. Many studies have implicated immune and inflammatory processes in the development of depression and antidepressant response [44, 64–71]. For example, Jansen et al. showed high expression of IL-6 signaling genes in MDD patients, and Wittenberg et al. demonstrated that anti-IL-6 and anti-IL-12/23 antibodies improve depressive symptoms in patients with inflammatory or oncological disorders [44, 72]. Of the immune pathways identified, TLR-related pathways were the most enriched in our meta-analysis. TLRs are present in microglia and other glial cells, and their activation leads to the release of cytokines and other inflammatory responses. Rodents exposed to stress or glucocorticoid administration have been shown to exhibit increased levels of TLR2 and TLR4, while blockade of TLR2 and TLR4 has been shown to prevent neuroinflammatory response [71, 73–76]. Additionally, one study included in our meta-analysis reported inflammation via the TLR pathway in depression pathogenesis and investigated the use of acupuncture in relieving this inflammation [55]. The consistent enrichment of the NF-κB signaling pathway upon meta-analysis is connected to this pattern, as activity of this pathway has been shown in rodents to mediate depressive-like behavior and increase release of pro-inflammatory cytokines in the microglia [77].

However, the direction of immune enrichment across the meta-analyzed studies was not consistent: while immune pathways were strongly upregulated in resistant samples in the two studies profiling gene expression in blood (including one study in human patients), these pathways were weakly upregulated in responding mice in studies profiling brain tissues. A recent systematic review has noted that inflammatory markers are only weakly correlated between peripheral and cerebrospinal fluid [78]. Carillo-Roa et al. identified 85 differentially expressed genes (q < 0.05) in the blood of responders vs. non-responders in mice treated with paroxetine, but similar differential expression was not observed in mouse prefrontal cortex; importantly, they demonstrated that these 85 genes could correctly predict response with 81% accuracy in humans treated with duloxetine or escitalopram, demonstrating that transcripts found in blood may be an accessible, objective diagnostic marker even if expression patterns are not shared in brain [79]. Additionally, Le-Niculescu et al. have identified biomarkers for mood disorders that are present in both brain and blood, providing more evidence for the value of these markers [20].

Protein metabolism pathways were the most consistently upregulated in samples resistant to fluoxetine treatment across organisms and tissue types. Specifically, two pathways involving the 40S and 60S ribosomal subunits were enriched; a few studies have previously identified ribosomal proteins and pathways as implicated in response to antidepressants [80, 81]. Additionally, Zhou et al. have reported evidence that ribosomes regulate gene expression involved in immune response [82].

Tissue heterogeneity again played a role when considering gene expression changes due to fluoxetine treatment, with blood-derived tissues showing distinct overall effects from those in the brain (Fig. 5E). However, considering the 55 widely varying comparisons of fluoxetine treatment vs. control, 17 pathways were identified as consistently differentially expressed with the Max-P method (Fig. 5G). Of these, GPCR binding pathways were most consistently downregulated by fluoxetine according to Max-P meta-analysis, and the GABAergic synapse was most downregulated according to vote. GABAergic neurons have been implicated in depression and antidepressant response, and positive modulators of the GABA_A_ receptor have been approved by the FDA for postpartum depression [83–86]. Additionally, we identified *Bdnf* as most consistently upregulated with treatment in stressed mice (q«0.001 by Max-P, upregulated with *p* < 0.05 in all seven studies); Tanaka et al. observed that *Bdnf* inhibits the GABA_A_ synaptic response in rat hippocampus [87]. Evidence from rodent studies has also suggested bidirectional connections between *Bdnf* expression and inflammation, as inflammation via lipopolysaccharide treatment was shown to increase *Bdnf* secretion in the microglia, while application of *Bdnf* in spinal cord injury has been shown to decrease microglial density [88–90]. Other studies have demonstrated similar connections in human and rodent models, indicating that elevation of BDNF by fluoxetine may participate in amelioration of depression symptoms by both GABAergic and anti-inflammatory effects [91–93].

When considering only stressed models, many more pathways (110) were identified as consistently affected by fluoxetine treatment (Fig. 6A), and interaction analysis of one study identified 48 pathways as differentially regulated by fluoxetine treatment in stressed vs. unstressed rodents, suggesting broader modulation of cellular processes under stressed conditions. Stressed models are likely more relevant in understanding the mechanism of action of antidepressants in MDD treatment, although naïve models have been reported to isolate pharmacological effects without the potentially confounding influence of stress [4, 60, 94]. Further separation into treatment effects of responders and non-responders demonstrated a greater number of pathways consistently affected by treatment in responders than non-responders (Supplementary Fig. 14). Effects were again observed in signal transduction and immune pathways; multiple signal transduction pathways were also identified as upregulated by duloxetine treatment in a larger patient cohort, in addition to BCR signaling, which was identified as upregulated in responders in our meta-analysis by Max-P and upregulated in remitted MDD patients in a naturalistic study [44, 45]. Pathways related to cancer were also identified in responders; antidepressants have recently been demonstrated to inhibit liver and lung cancer through the mTOR pathway, although other evidence has indicated associations between antidepressant use and increased cancer incidence [95–97].

While the PRISMA guidelines are more commonly applied for systematic review of clinical studies, we felt that they provided a strong framework for this work. Systematic identification and re-analysis of relevant data sets allowed us to calculate consistent metrics for differential expression, rather than relying on lists of statistics provided by individual study authors. We used broad inclusion criteria for organism and tissue to determine whether consistent changes were observable across heterogeneous studies; the use of Fisher’s meta-analysis method, subgroup analyses, and sensitivity analyses then let us explore the expected heterogeneity. Reporting bias is challenging to assess in gene expression analysis, where thousands of statistics are generated for each study. Yousefi et al. have assessed risk of reporting bias when classification algorithms are applied to gene expression data [98], but we are not aware of methods to detect reporting bias of the gene expression data itself; this will be a valuable tool as systematic reviews of gene expression studies become more prevalent.

This study did include multiple limitations that should be addressed to better understand antidepressant effectiveness. Only two studies provided gene expression data for responders and non-responders prior to treatment, so it is not possible to derive strong inferences for predictive markers of fluoxetine response; even large-scale patient studies have struggled to identify general predictive markers to this point [15, 99]. Only two patient cohorts were included in our meta-analyses: while other studies have been conducted in patients, they tend to focus on a small number of biomarkers or otherwise do not submit full expression data to GEO [15]. Most data sets profiled in our meta-analyses contained sample sizes of five or fewer per group, although some samples were pooled across multiple rodents. We thus compared our results with two studies of larger patient cohorts (one with duloxetine and one naturalistic study comparing patients with remitted vs. current MDD): we observe some overlap between the results which support our general findings, but we note that these studies contain obvious differences from those included in our meta-analyses, in addition to less obvious differences that we may not have identified [44, 45]. Additionally, this systematic review was not prospectively registered, and the reviewers did not work independently (all exclusion decisions are documented in Supplementary Table 1).

The included studies showed substantial bias based on sex, with 17 of 18 rodent studies profiling males, while the two patient studies included majority or exclusively female participants; approximately 70% of participants were female in the two larger-cohort studies used for comparison. Results of Max-P meta-analysis provide some evidence for conserved response and treatment signatures, but it is not possible to identify sex-dependent biological signatures with these data. Previous work has shown both consistency and divergence between males and females regarding clinical response, molecular signatures, and adverse effects from antidepressants, and this remains an important area of study [42, 100–103].

Considering the analysis methods, pre-ranked pathway analysis methods have been criticized as overoptimistic because they ignore gene-gene correlations, although recent work suggests that these simple tools have power to identify relevant biological insights [104–107]. We applied the stringent Max-P method to identify those consistently identified across studies—additionally, we compared the meta-analysis of results obtained by GSEA to those obtained from the DAVID functional annotation tool, and saw good concordance for Fisher’s meta-analysis (but we note that no pathways were identified by Max-P from DAVID analysis). Our reliance on the Max-P method for meta-analysis means that we are particularly sensitive to a single aberrant study resulting in high p-values due to data quality or an unconsidered factor, which may not have been apparent during screening. For this reason, all code and results are provided in our registered repository for further inspection and analysis, including individual data set analyses, as well as other meta-analyses by Fisher’s method (which is more sensitive to studies that may have extremely low p-values) and the Freq-50 method (which may be most robust but does not test a specific hypothesis). We hope that other researchers access and analyze these data in other ways, facilitating more detailed discoveries for further validation.

Our meta-analyses have emphasized some known pathways in antidepressant response and unearthed a few new routes of potential investigation, but a true understanding of antidepressant response will require additional large-cohort studies focusing on transcriptomic data. However, recent studies of antidepressant response in over 100 patients have resulted in few, if any, individual biomarkers of response, supporting the consensus that there is not a single genetic signature of response, but a variety of contributing ‘omic and environmental factors [15, 99]. Emerging technologies and approaches have begun to allow us to understand genomics and transcriptomics at a deeper level, including single-cell profiling, alternative splicing, and epigenetic factors [60, 108–110]. And as technology improves our ability to quantify protein and metabolite levels, it will be valuable to incorporate these data, as this will bring us even closer to the true biology underpinning these complex disorders.

## Supplementary information

Supplementary Information

Supplementary Table 1

Supplementary Table 2

Supplementary Table 3

## Data Availability

All analyses completed in this work are included at https://github.com/DavidGCooper/FLX-MetaDE with 10.5281/zenodo.10668845 [111].
