# Supplementary material for: Gene expression signatures of response to fluoxetine treatment: systematic review and meta-analyses
Source: Mol Psychiatry. 2025 Jul 17;30(11):5563–77. doi: 10.1038/s41380-025-03118-6 (PMC12532588; doi:10.1038/s41380-025-03118-6)
Supplement: Supplementary file 1 — Supplementary Information [file 41380_2025_3118_MOESM1_ESM.docx]

*Supplementary Materials for:*

Gene expression signatures of response to fluoxetine treatment: systematic review and meta-analyses

David G. Cooper, Ph.D.; J. Paige Cowden, Pharm.D.; Patrick M. Vo; Parker A. Stanley; Jack T. Karbowski; Victoria S. Gaertig; Caiden J. Lukan, Pharm.D.; Ariel D. Worthington, Pharm.D. ; Caleb A. Class, Ph.D.*

Contents

[*Supplementary Methods* 2](#_Toc183441903)

[*Supplementary Results* 3](#_Toc183441904)

[Subgroup analyses for response signatures 3](#_Toc183441905)

[Sensitivity analyses for response signatures 4](#_Toc183441906)

[Interaction analysis of treatment effects in stressed and unstressed mice 5](#_Toc183441907)

[Subgroup analyses for treatment signatures 5](#_Toc183441908)

[Sensitivity analyses for treatment signatures 6](#_Toc183441909)

[*Supplementary References* 7](#_Toc183441910)

[*Supplementary Figures* 9](#_Toc183441911)

# *Supplementary Methods*

Studies were synthesized across organisms, and across studies profiling the same organism, using the meta-analyses described in **Methods**. For subgroup analyses within organisms, p-values were synthesized at both the gene and pathway level, while across-organism syntheses were only conducted at the pathway level (described in **Results**). Additional syntheses of treated vs. untreated comparisons were conducted within the subgroup of studies profiling depressed patients or stressed rodents, and separately across studies profiling unstressed rodents. Finally, we conducted one interaction analysis to identify genes differentially affected by fluoxetine treatment in stressed vs. unstressed mice, using two datasets from the same study that profiled fluoxetine effects in both stressed and unstressed mice in dentate gyrus, cingulate gyrus, and whole blood (1). Interaction analyses were conducted at the gene level in limma, and differentially affected genes were summarized to the pathway level using fgsea with the same pathway databases as before (2,3). Meta-analysis was conducted to synthesize the interaction analyses across the three tissue types.

Sensitivity analyses were performed to assess the meta-analyses, as described in the PRISMA guidelines (4). Of nine response vs. non-response comparisons, eight involved mice while the other included human patients, so we looked at inclusion/exclusion of the patient data set. We further explored the effect of tissue type by removing the single comparison profiling blood in mice, resulting in seven comparisons in brain tissue only. Additionally, we compared meta-analysis results of treatment signatures when DS19 (containing 27 comparisons from various brain regions in unstressed rats) was removed from our meta-analysis (5).

We also assessed sensitivity of the results to both the statistical gene set analysis method and to the selection of gene set database, by comparing our GSEA results with those obtained using the DAVID Functional Annotation Tool with the default gene sets, which includes KEGG Pathways, UniProt Key Words, Gene Ontology, and others (6–9). For each DAVID analysis, we included genes with nominal p<0.05 by differential expression as our gene list, and all genes quantified in the study as the background list; the analysis was then run using the browser-based DAVID Functional Annotation Tool using the default gene sets. We then used the same meta-analysis methods as before to synthesize the DAVID results across studies, and we compared these results to those obtained from GSEA – in particular, we correlated the meta-analysis statistics calculated for the KEGG pathways, which were analyzed using both GSEA and DAVID.

# *Supplementary Results*

## Subgroup analyses for response signatures

Meta-analysis was conducted for differential expression at both the gene and pathway level for the eight comparisons within mice. More pathways were identified as consistently differentially expressed by Max-P (30 with only mouse studies vs. 18 including the patient cohort), although many of the uniquely identified pathways also involved TLR cascades (**Supplementary Figure 3**). Individual gene meta-analysis proved less consistent, with only 40 genes identified as differentially expressed in *any* comparison by Fisher’s method, four differentially expressed in over half of the comparisons, and none identified as consistent by the Max-P method (**Supplementary Figure 4A**). A meta-volcano plot of these results is presented in **Supplementary Figure 4B**. *Fosl2* is both highly enriched by Fisher’s method (q<0.001) and most consistently upregulated in good responders (vote sum of +5). *Notch4* (+4), *Golm1* (-4), and *Adamts* (-4) are the other genes that are most consistently differentially expressed across comparisons in mice.

Additionally, we compared results from two studies that profiled gene expression in responders and non-responders that had not previously received fluoxetine to identify potential predictive signatures: one in patient-derived LCL’s (DS3) and the other in mouse cortex samples (DS1, mouse response was inferred based on overall response measured for multiple mice from each strain) (10,11). 13 pathways were identified as enriched by GSEA with q<0.05 in both comparisons, and all were upregulated in good responders (**Supplementary Figure 5A**). These included pathways related to metabolism and mitochondrial translation, in addition to gene signatures of Alzheimer’s disease and Parkinson’s disease. Ten of these pathways were also enriched (but upregulated in non-responders) in the larger-cohort study by Belzeaux *et al.* for responding vs. non-responding patients profiled prior to duloxetine treatment (**Supplementary Figure 5B**) (12).

## Sensitivity analyses for response signatures

We conducted two sensitivity analyses successively removing individual comparisons from the meta-analysis for responders vs. non-responders. The previously presented meta-analysis across mouse studies demonstrated the effects of removing the single patient data set; we then conducted a second analysis synthesizing the seven mouse comparisons in brain tissues only (**Supplementary Figure 3**). As individual data sets were removed, pathways identified by Fisher’s method decreased, as this method identifies whether a pathway is enriched in *any* of the studies. By Max-P, 12 additional pathways were identified when the patient comparison was removed (as previously discussed), and one more (LDL clearance) was identified when only the seven brain comparisons were included. Thus, we can conclude that the Max-P meta-analysis of responders vs. non-responders was only slightly sensitive to the inclusion of gene expression profiling in blood samples, as all pathways identified as consistently affected in brain were also affected in blood (although not necessarily in the same direction).

Additionally, we compared the results of our pathway meta-analysis to those obtained with the DAVID functional annotation tool, which conducts over-representation analysis based on a list of differentially expressed genes rather than analysis of a ranked list of genes, followed by the same meta-analysis methods. Zero pathways were identified by Max-P as consistently enriched with differentially expressed genes, and four pathways were identified using Fisher’s method (compared with 357 from meta-analysis of GSEA results): GO terms for protein binding and the extracellular region, the UniProt Keyword for Calcium, and COMPBIAS Polar Residues. The low number of enriched pathways by DAVID analysis is likely due to its reliance on the few differentially expressed genes identified within individual studies for response comparisons (by contrast, many more differentially expressed genes are identified within treatment vs. control comparisons), as GSEA was able to identify patterns of small but concerted differential expression among pathways.

## Interaction analysis of treatment effects in stressed and unstressed mice

Two datasets from the same study profiled fluoxetine effects in both stressed and unstressed mice in dentate gyrus, cingulate gyrus, and whole blood (1). We conducted interaction analysis to identify genes differentially affected by fluoxetine treatment between groups, and then estimated pathway enrichment with GSEA before meta-analysis across tissues by MetaDE. 48 pathways were consistently identified as differentially affected by fluoxetine treatment between stressed and unstressed mice in all three tissues (q<0.05 by Max-P meta-analysis). Some overlap was observed with the overall meta-analysis results for treated vs. untreated subjects, as one pathway related to nonsense-mediated decay was identified as altered within both stressed and unstressed subjects (generally downregulated by fluoxetine), and nine other pathways, largely involved in RNA or protein metabolism, were identified in one meta-analysis but not the other (**Supplementary Figure 9**). Additionally, 3 protein metabolism pathways identified in interaction analysis were also significant by Max-P meta-analysis in comparison of responding vs. non-responding subjects.

## Subgroup analyses for treatment signatures

A summary of within-species meta-analysis results is presented in **Supplementary Figure 12**. Profiling treatment effects in stressed mice identified the greatest number of differentially expressed genes, with 11,541 identified by Fisher’s method and 1729 identified as consistently differentially expressed by Max-P (**Supplementary Figure 13A**). Genes identified by both Fisher’s method and Max-P had a wider distribution of vote sums, with 39.8% with absolute value of 3 or greater (**Supplementary Figure 13B**). Seven genes were significantly affected by treatment in the same direction across all seven comparisons: *Bdnf, Zfp703, Raogap1, Rcan1, Dock10, St8sia4,* and *Lrrn3* (**Supplementary Figure 13C**). Of these, Brain-derived neurotrophic factor (*Bdnf*) was identified as most consistently upregulated by Max-P (q<<0.001).

Additionally, we conducted separate analyses and meta-analyses within responder and non-responder groups across studies that provided response information, to identify gene expression changes that may indicate response to treatment. The Max-P method identified 20 pathways that were consistently changed across both groups, 84 pathways that were specific to responders, and 29 that were specific to non-responders (**Supplementary Figure 14A**). Of the pathways that consistently changed with treatment in responders, we focused on the 15 that were consistently *unchanged* in non-responders (**Supplementary Figure 14B**, q>0.05 in non-responders by Max-P and Fisher’s method). This included six pathways from the KEGG database, four signal transduction pathways, two immune pathways, and three others. However, vote sums ranged from -2 to +2 with one exception (AGE-RAGE signaling in diabetic complications, +4), indicating weak certainty of evidence.

## Sensitivity analyses for treatment signatures

We conducted a sensitivity analysis removing DS19 (profiling across 27 brain regions, some of which were reported as minimally affected by fluoxetine, in unstressed mice), as this was about half of the comparisons included in the full synthesis of all studies comparing treated vs. untreated patients or rodents (5). We expected our Max-P results to be sensitive to comparisons in less-affected brain regions—surprisingly, we saw that fewer pathways were consistently enriched upon removal of this study (**Supplementary Figure 15**). Considering the gene level, 148 genes were consistently differentially expressed in the original meta-analysis, vs. none when DS19 was removed. Thus, we can conclude that meta-analysis of treatment effects was sensitive to DS19. As seen in **Figure 4E**, comparisons across the 27 tissue types in DS19 showed largely consistent patterns, likely contributing to the observed result.

Next, we compared our meta-analysis results to those obtained when DAVID was employed rather than GSEA. No gene sets were identified as consistently altered by fluoxetine across depressed patients and stressed rodents by Max-P, although six demonstrated enrichment trends with q<0.2, including the GO term for cerebral cortex development, actin filament binding, and four gene sets related to EF-hand domains, which are important in calcium ion binding. Additionally, 434 gene sets were identified as enriched by Fisher’s meta-analysis with q<0.05, and 42 were enriched in at least half of comparisons according to Freq50; in particular, GO terms for protein binding, postsynapse, glutamatergic synapse, and dendrites were most frequently enriched by DAVID analysis (**Supplementary Table 3**). Comparison of Fisher’s meta-analysis results for KEGG pathways analyzed using both GSEA and DAVID revealed moderate agreement (Spearman’s rho of 0.47), with the most enriched gene sets related to Parkinson’s and Alzheimer’s disease, ribosome, and oxidative phosphorylation (**Supplementary Figure 16**). Additionally, analysis by DAVID identified olfactory transduction as the most enriched KEGG pathway for meta-analysis across all treated vs. untreated comparisons; this was also identified across studies using GSEA (q < 0.001), but to a lesser extent than with DAVID. We conclude that there is reasonable concordance between GSEA and DAVID in this case, as there were sufficient numbers of differentially expressed genes to identify enriched pathways using either over-representation analysis via DAVID, or ranked analysis via GSEA.

# *Supplementary References*

1. Hervé M, Bergon A, Le Guisquet AM, Leman S, Consoloni JL, Fernandez-Nunez N, et al. Translational Identification of Transcriptional Signatures of Major Depression and Antidepressant Response. Front Mol Neurosci. 2017 Aug 8;10:248.

2. Ritchie ME, Phipson B, Wu D, Hu Y, Law CW, Shi W, et al. limma powers differential expression analyses for RNA-sequencing and microarray studies. Nucleic Acids Research. 2015;43(7):e47.

3. Sergushichev A. An algorithm for fast preranked gene set enrichment analysis using cumulative statistic calculation. bioRxiv [Internet]. 2016; Available from: http://biorxiv.org/content/early/2016/06/20/060012

4. Page MJ, McKenzie JE, Bossuyt PM, Boutron I, Hoffmann TC, Mulrow CD, et al. The PRISMA 2020 statement: an updated guideline for reporting systematic reviews. BMJ. 2021 Mar 29;372:n71.

5. Rayan NA, Kumar V, Aow J, Rastegar N, Lim MGL, O’Toole N, et al. Integrative multi-omics landscape of fluoxetine action across 27 brain regions reveals global increase in energy metabolism and region-specific chromatin remodelling. Mol Psychiatry. 2022 Nov;27(11):4510–25.

6. Kanehisa M, Furumichi M, Tanabe M, Sato Y, Morishima K. KEGG: new perspectives on genomes, pathways, diseases and drugs. Nucleic Acids Research. 2017 Jan;45(D1):D353–61.

7. Sherman BT, Hao M, Qiu J, Jiao X, Baseler MW, Lane HC, et al. DAVID: a web server for functional enrichment analysis and functional annotation of gene lists (2021 update). Nucleic Acids Research. 2022 Jul 5;50(W1):W216–21.

8. The UniProt Consortium. UniProt: the Universal Protein Knowledgebase in 2023. Nucleic Acids Research. 2023 Jan 6;51(D1):D523–31.

9. Ashburner M, Ball CA, Blake JA, Botstein D, Butler H, Cherry JM, et al. Gene Ontology: tool for the unification of biology. Nat Genet. 2000 May;25(1):25–9.

10. Breitfeld J, Scholl C, Steffens M, Brandenburg K, Probst-Schendzielorz K, Efimkina O, et al. Proliferation rates and gene expression profiles in human lymphoblastoid cell lines from patients with depression characterized in response to antidepressant drug therapy. Transl Psychiatry. 2016 Nov 15;6(11):e950.

11. Benton CS, Miller BH, Skwerer S, Suzuki O, Schultz LE, Cameron MD, et al. Evaluating genetic markers and neurobiochemical analytes for fluoxetine response using a panel of mouse inbred strains. Psychopharmacology. 2012 May 1;221(2):297–315.

12. Belzeaux R, Gorgievski V, Fiori LM, Lopez JP, Grenier J, Lin R, et al. GPR56/ADGRG1 is associated with response to antidepressant treatment. Nat Commun. 2020 Apr 2;11(1):1635.

# *Supplementary Figures*


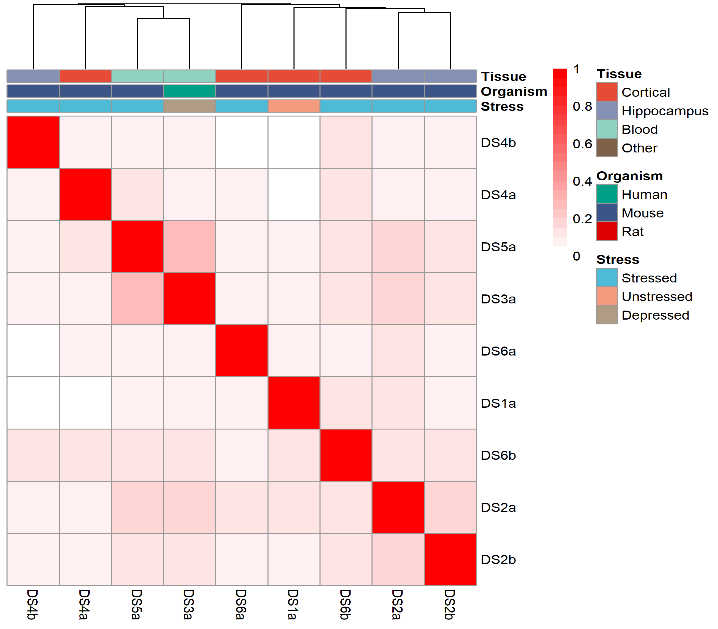

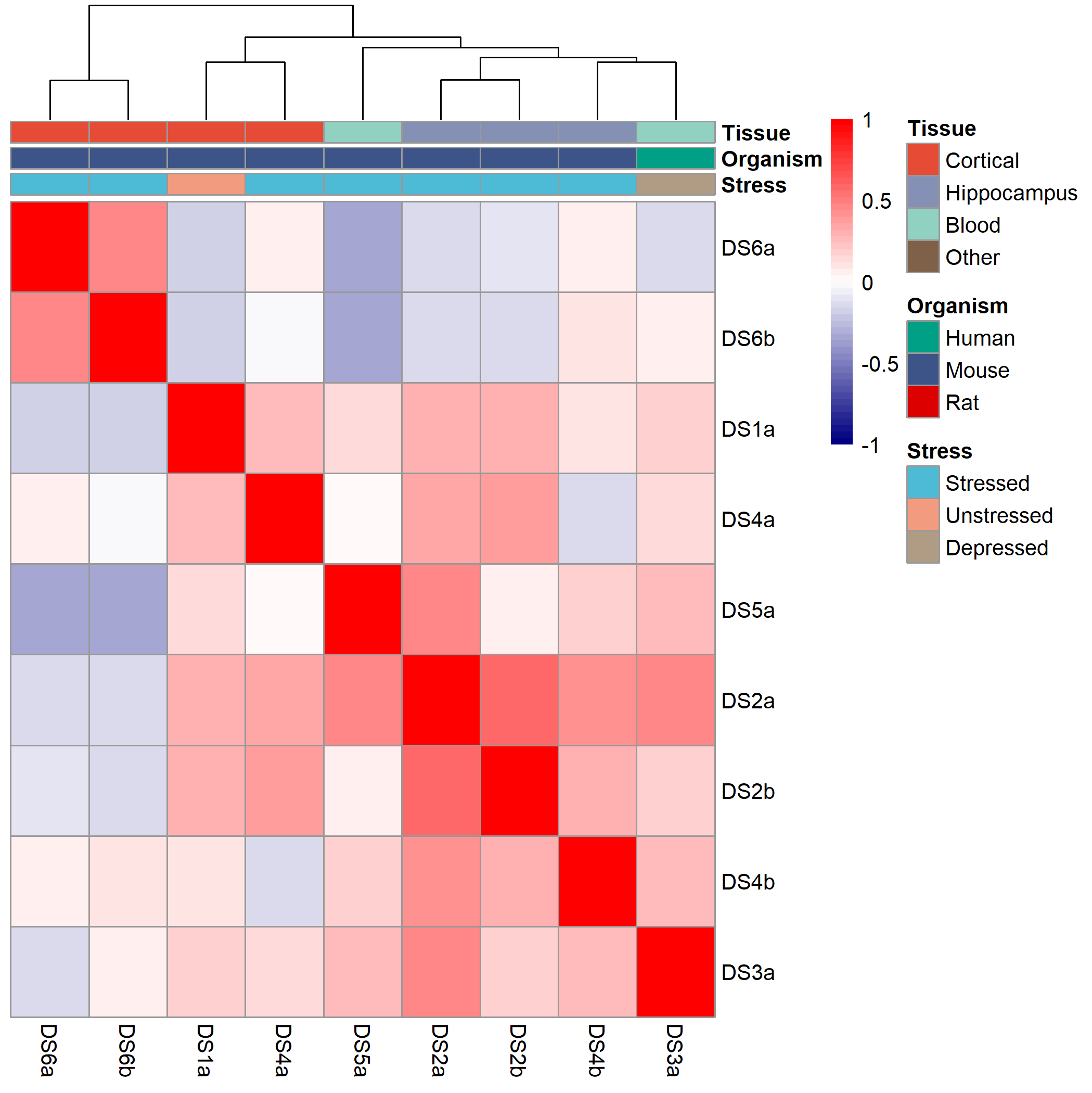


***Overlap***

***Correlation***

Supplementary Figure 1. Comparison of pathway enrichment results across comparisons of responders vs. non-responders. (*Left*) Overlap between studies of pathways enriched with nominal p<0.05, calculated with Jaccard index. (*Right*) Pearson correlations of Normalized Enrichment Scores.

**B.**

**A.**


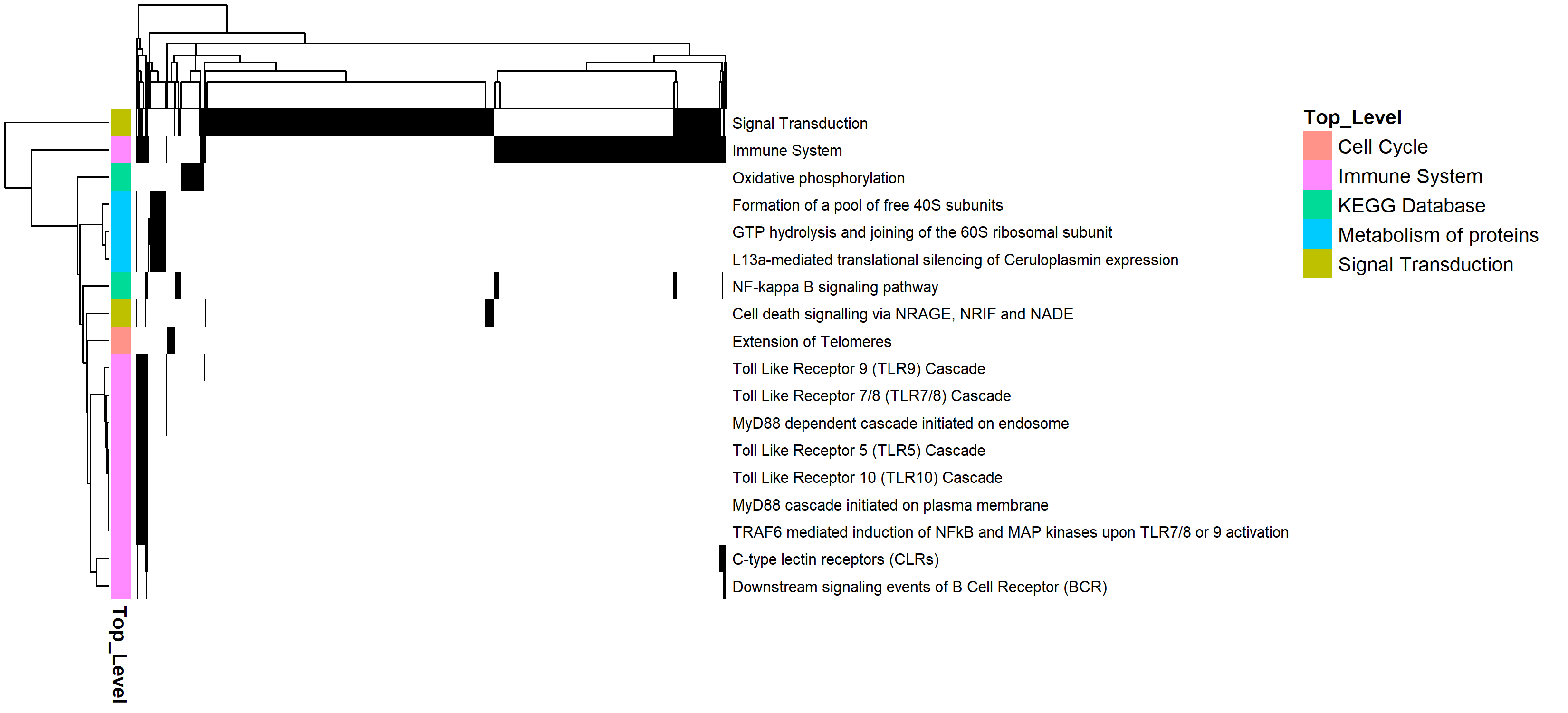

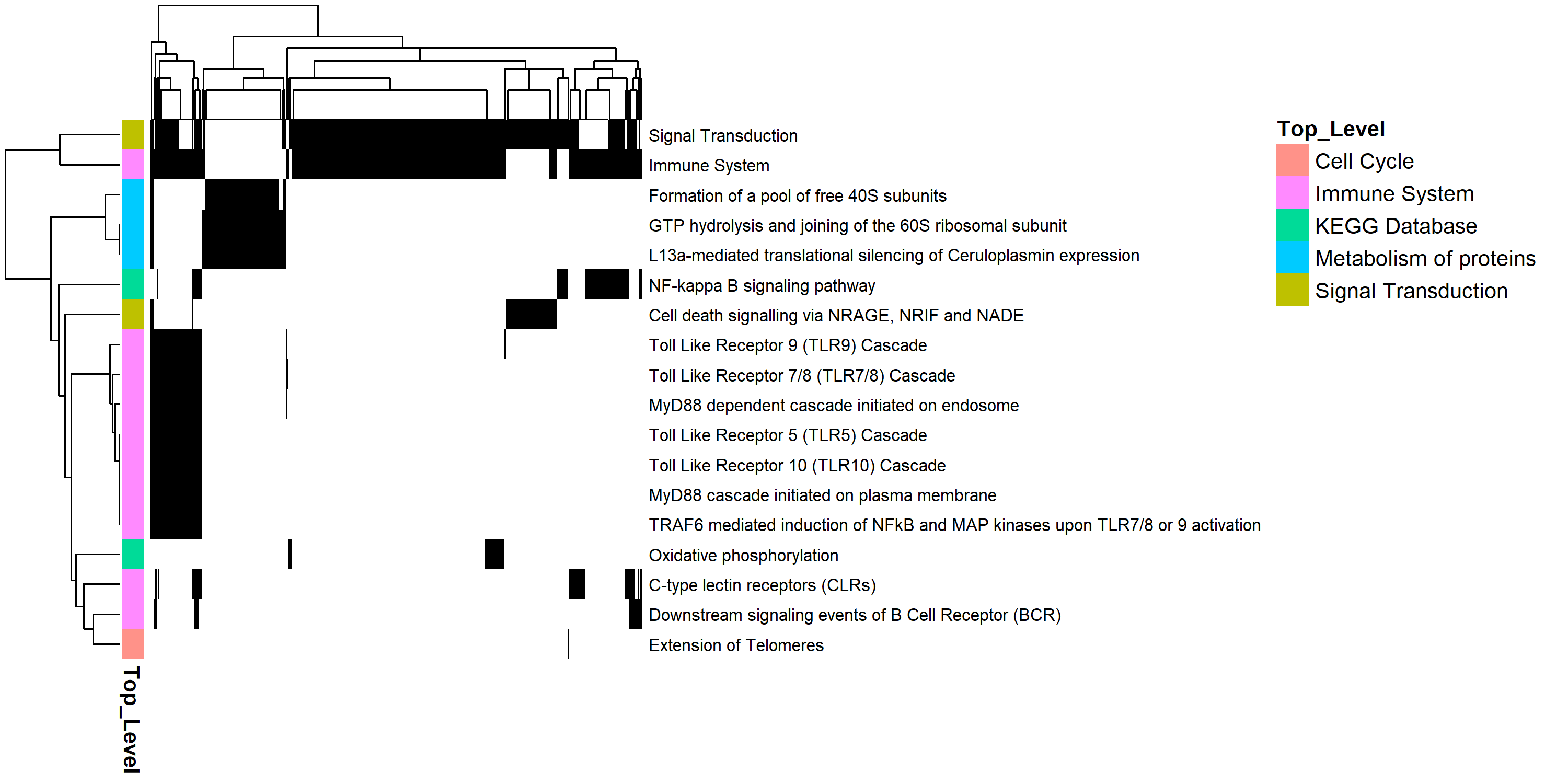


Supplementary Figure 2. Overlap of genes included in pathways identified as significant across the nine comparisons of responders vs. non-responders (q < 0.05 by Max-P). Rows of heatmap indicate pathways, columns indicate genes. Black fill indicates that a gene is a member of a certain pathway. (A) All included genes. (B) Subset of genes appearing in at least two of the pathways.


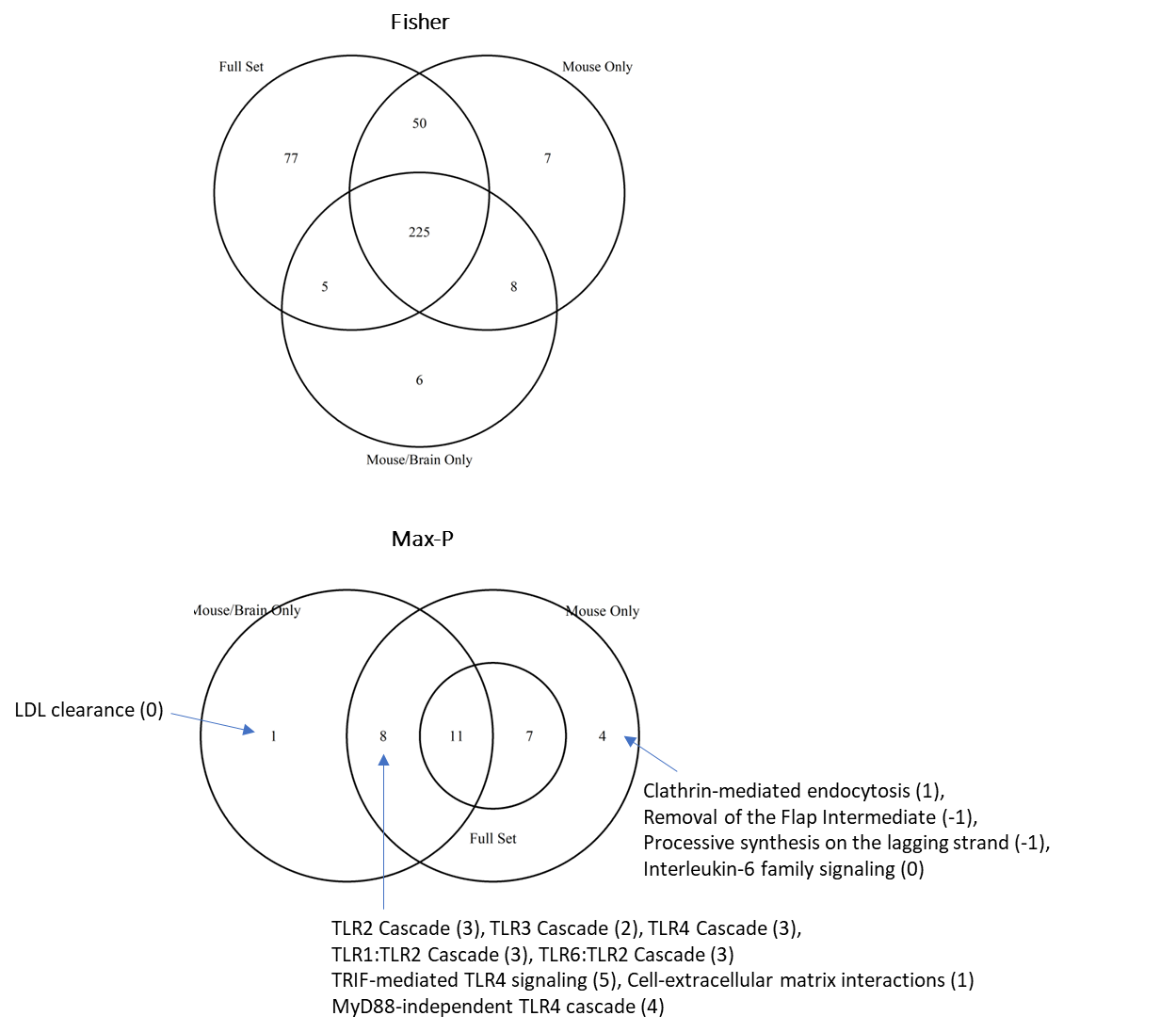


Supplementary Figure 3. Overlap of pathways identified for responders vs. non-responders for all nine comparisons, with two sensitivity analyses (meta-analysis with human comparison removed, and meta-analysis with blood comparisons removed). Vote sums are indicated in parentheses.


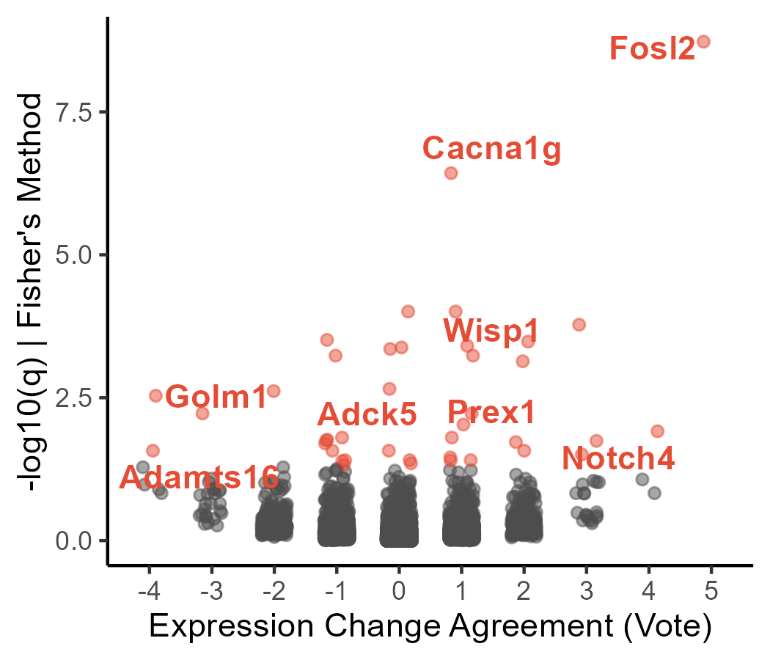


**A.**

**B.**


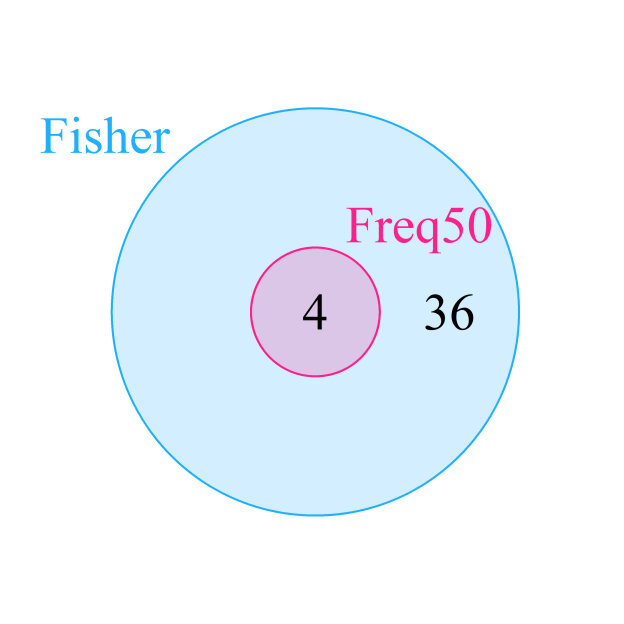


Adck5, Fosl2, Prex1, Wisp1

Supplementary Figure 4. (A) Meta-analysis results for genewise differential expression in eight mouse comparisons. No genes were identified as consistently differentially expressed by Max-P. (B) Volcano plot of mouse differential expression meta-analysis results. Red points indicate q < 0.05 by Fisher’s meta-analysis.


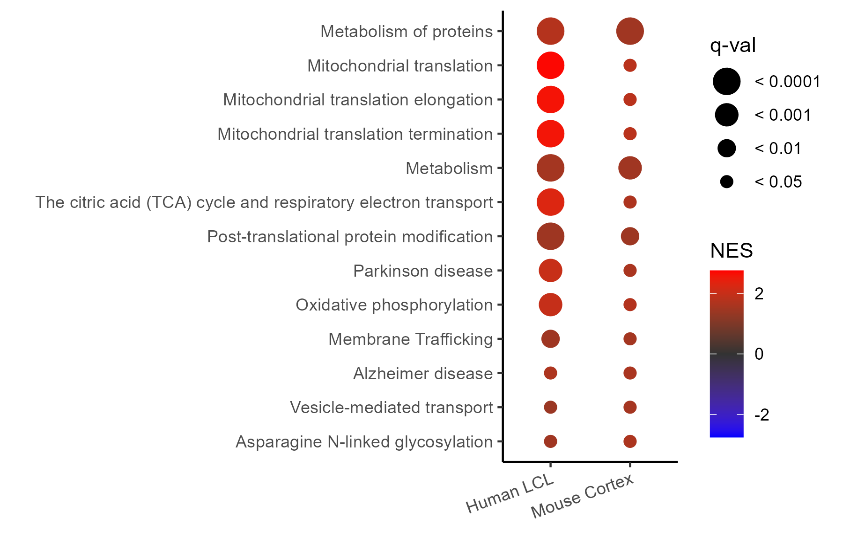


**A.**

**B.**


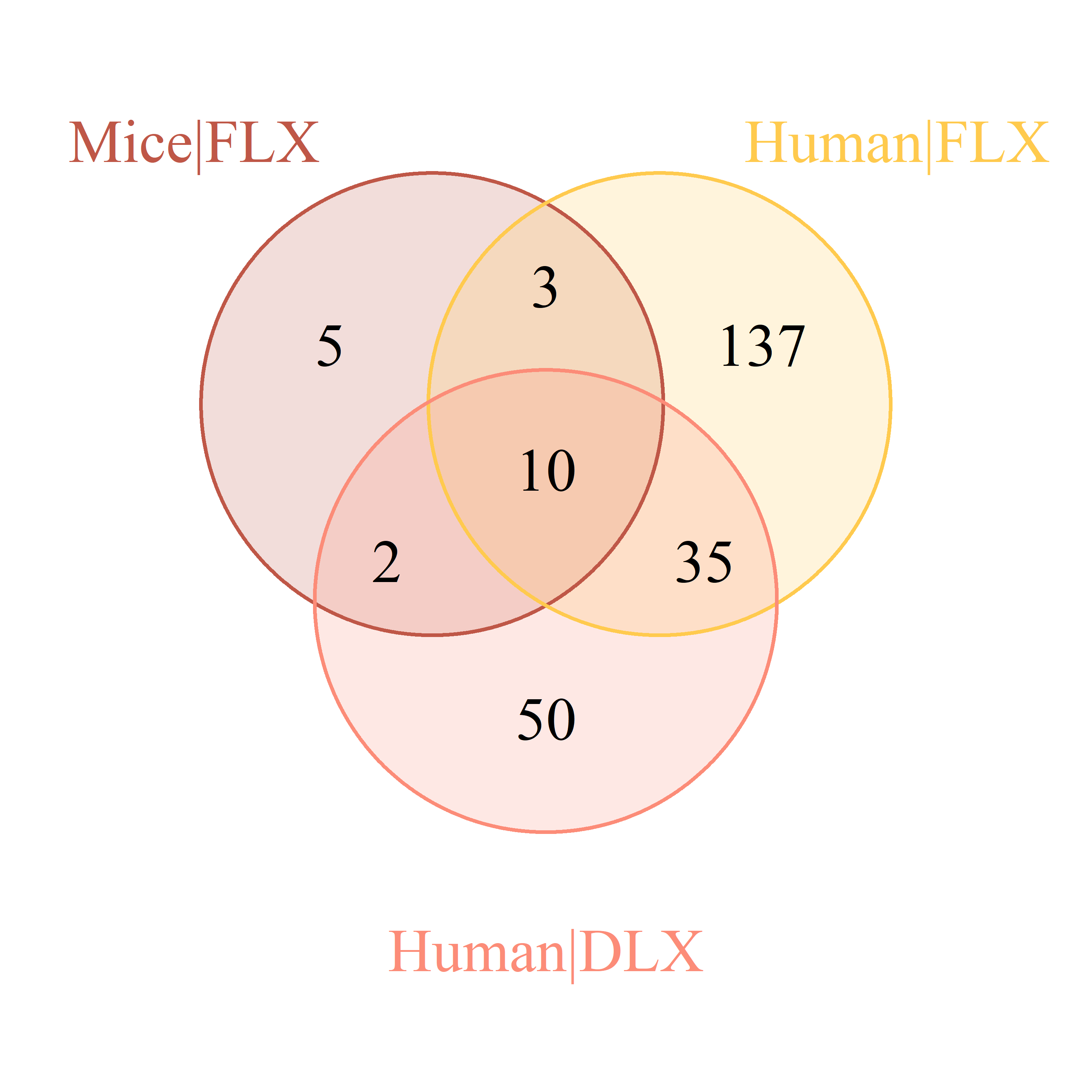


Supplementary Figure 5. (A) 13 pathways enriched in responders vs. non-responders in two comparisons of samples not treated with fluoxetine (q < 0.05 in each). (B) Overlap between pathways identified in two comparisons from (A), of samples prior to treatment with fluoxetine, and the study by Belzeaux *et al.* profiling patient blood prior to treatment with duloxetine to compare good vs. poor responders (“Human|DLX”).


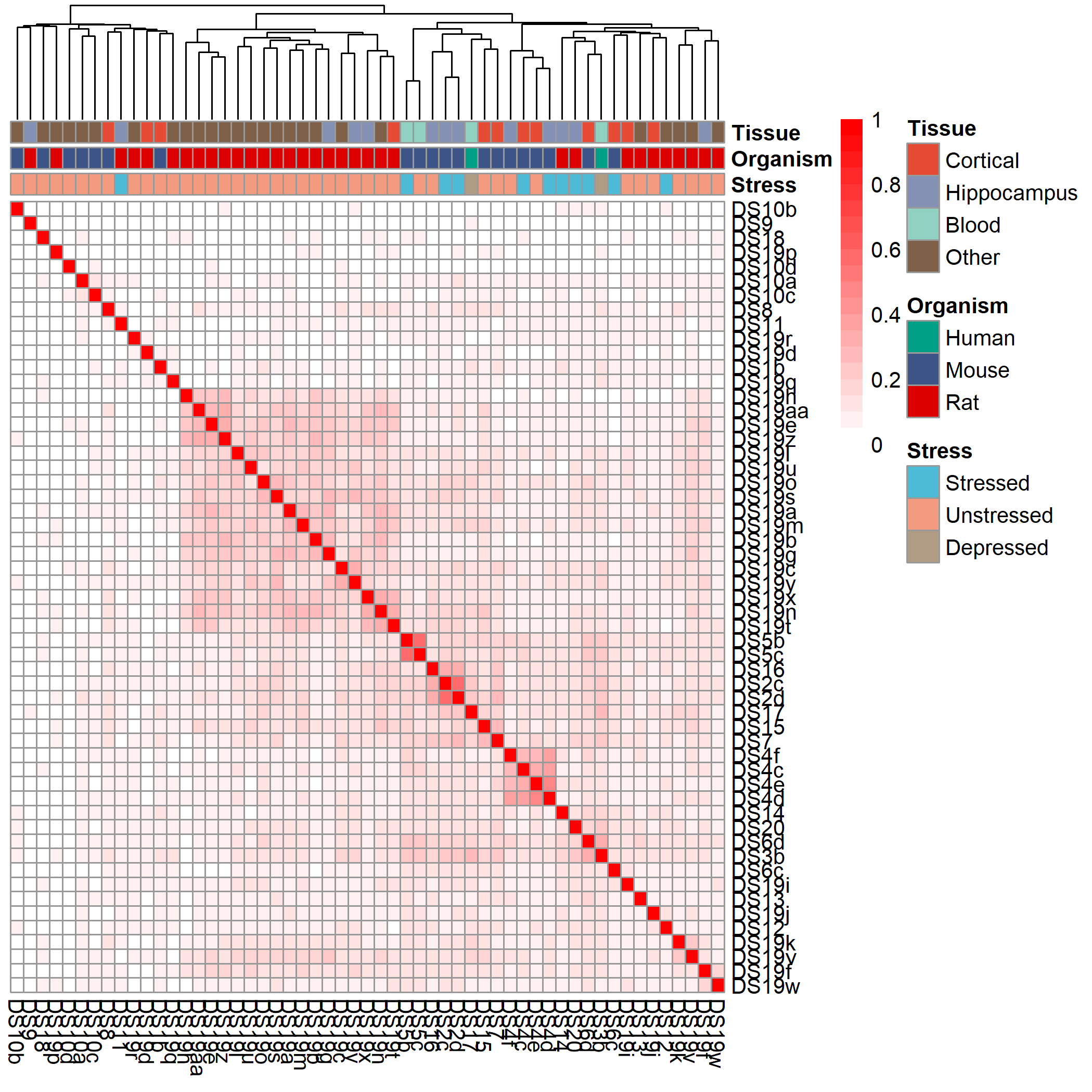


Supplementary Figure 6. Overlap between studies of pathways enriched with nominal p<0.05, across comparisons of treated vs. untreated samples. Overlap quantified by Jaccard Index.


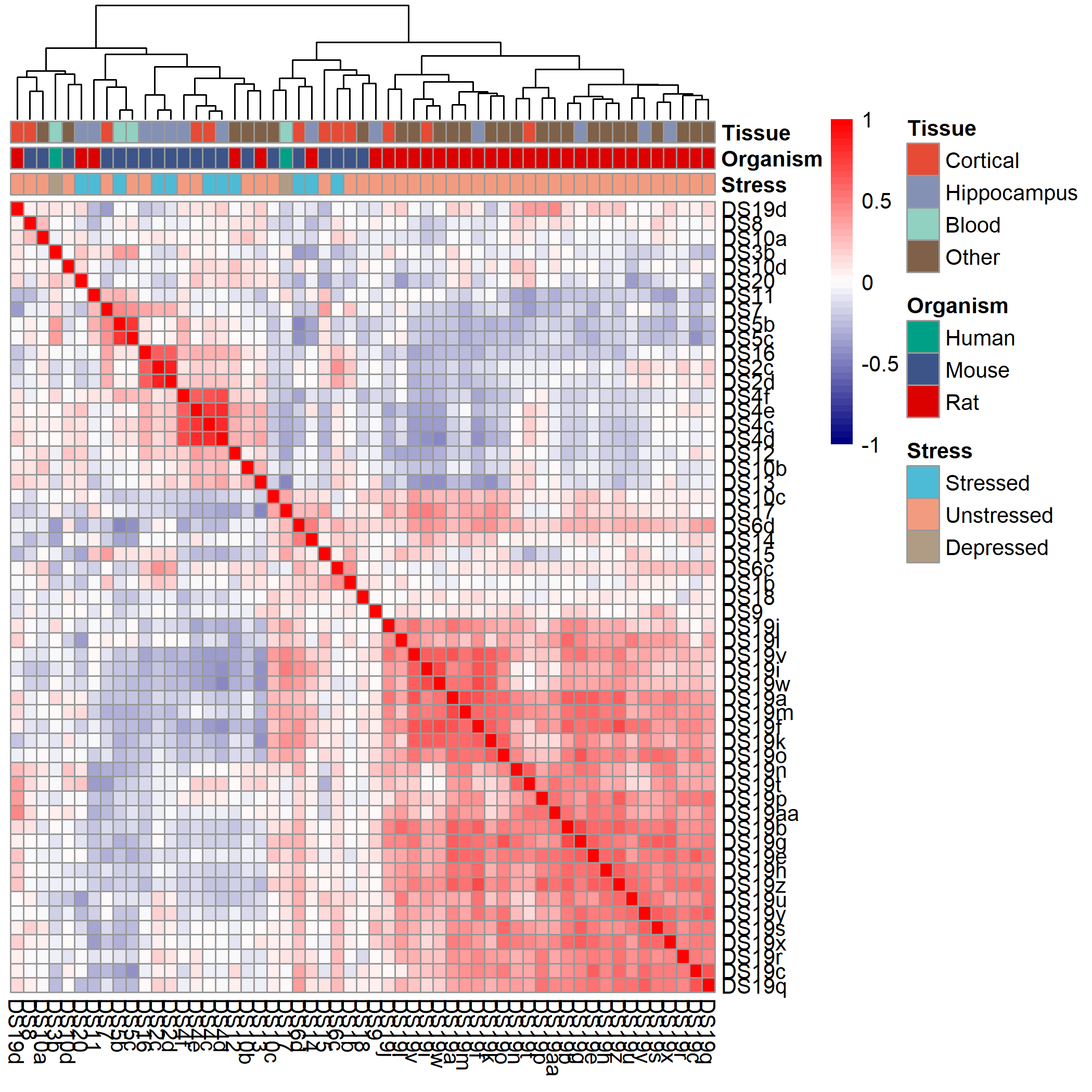


Supplementary Figure 7. Pearson correlation of Normalized Enrichment Scores across comparisons of treated vs. untreated samples.


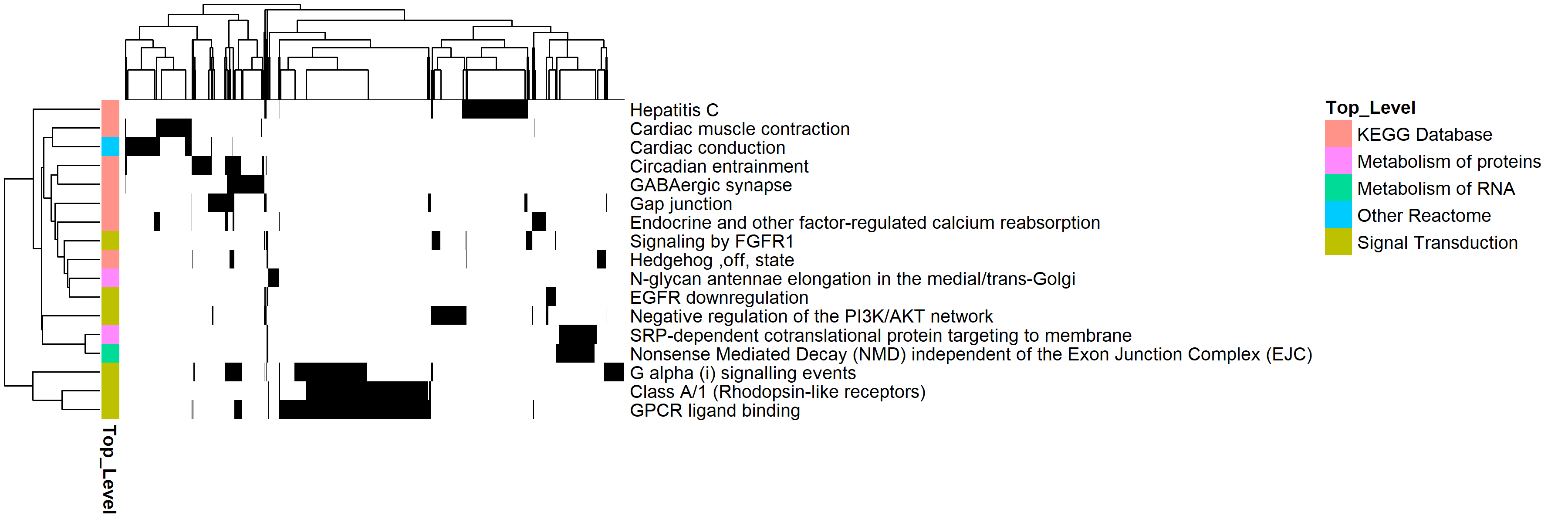


Supplementary Figure 8. Overlap of genes included in pathways identified as significant across all comparisons of fluoxetine treated vs. untreated samples (q < 0.05 by Max-P). Rows of heatmap indicate pathways, columns indicate genes. Black fill indicates that a gene is a member of a certain pathway.


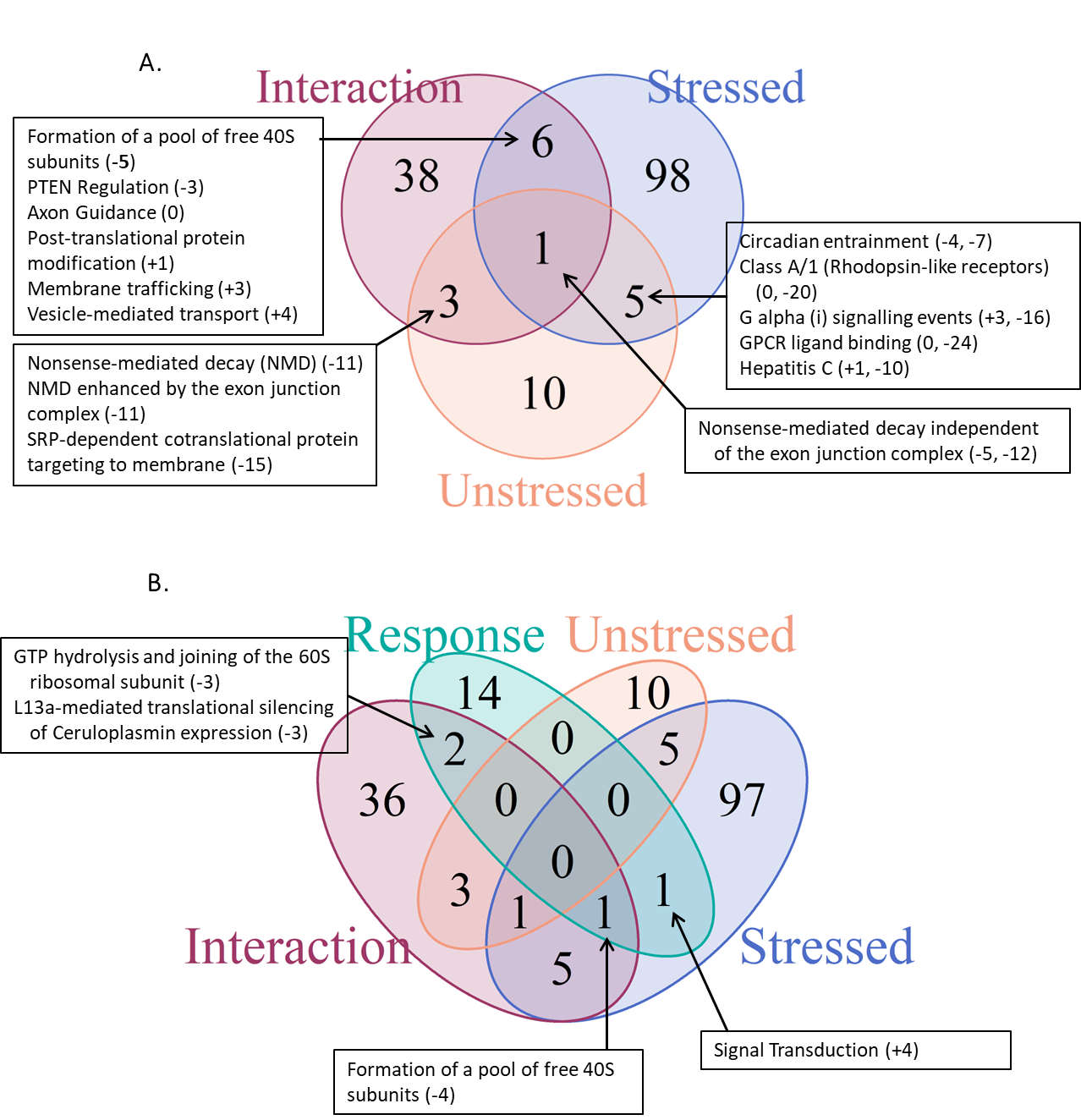


Supplementary Figure 9. (A) Overlap of pathways identified as statistically significant by Max-P meta-analysis of treated vs. untreated samples in stressed rodents or depressed patients, in unstressed rodents, and pathways identified by interaction analysis comparing fluoxetine effects in stressed vs. unstressed mice. Vote sums are indicated for the relevant meta-analysis; if identified by both meta-analyses, they are indicated as (stressed, unstressed). (B) Same overlap, with addition of pathways identified by Max-P for responders vs. non-responders (“Response”, with Vote sums indicated in parentheses).


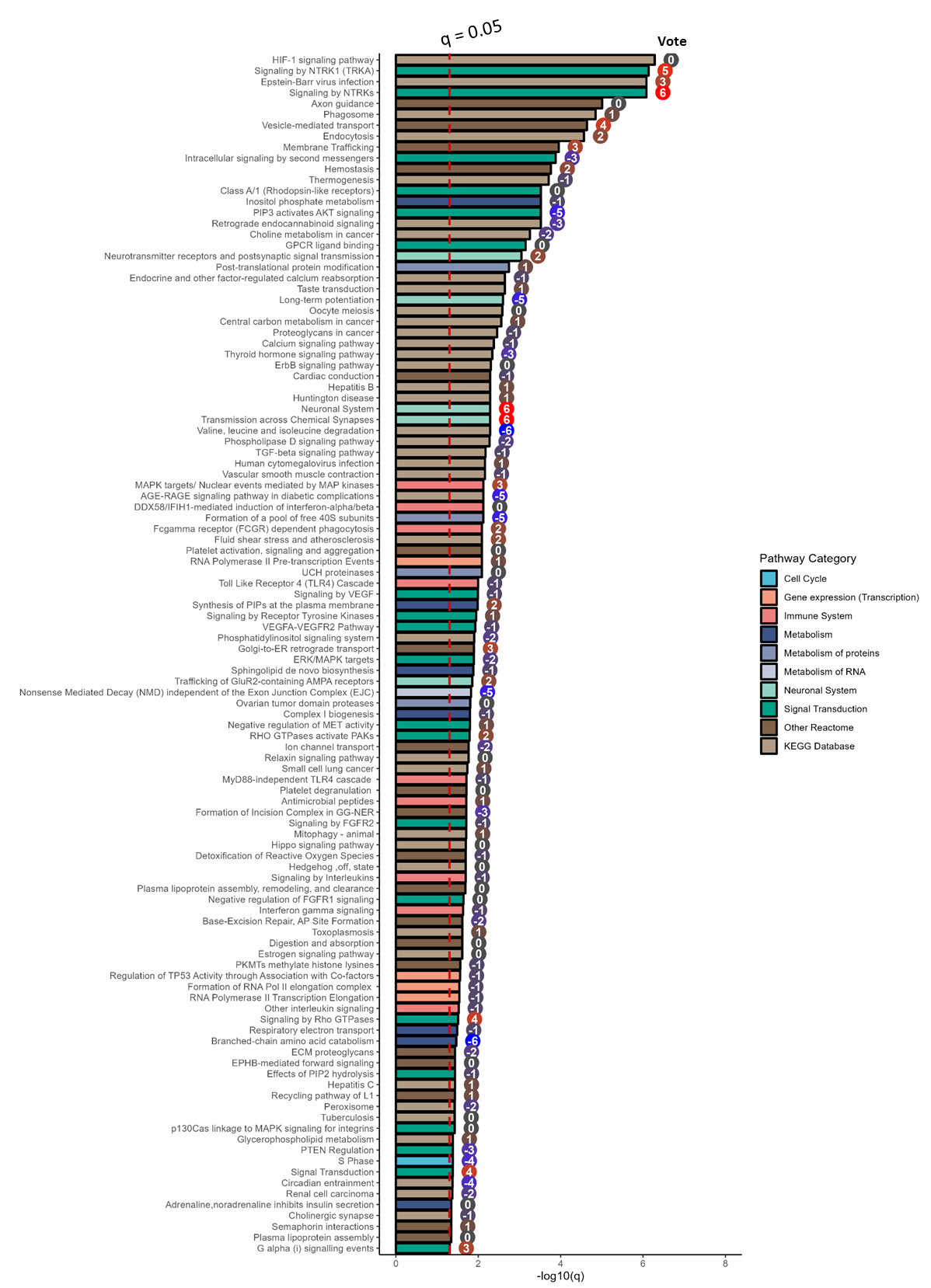


Supplementary Figure 10. q-value by Max-P and Vote Sums for all pathways identified as significant across the 13 comparisons in MDD patients or stressed rodents (q < 0.05 by Max-P).


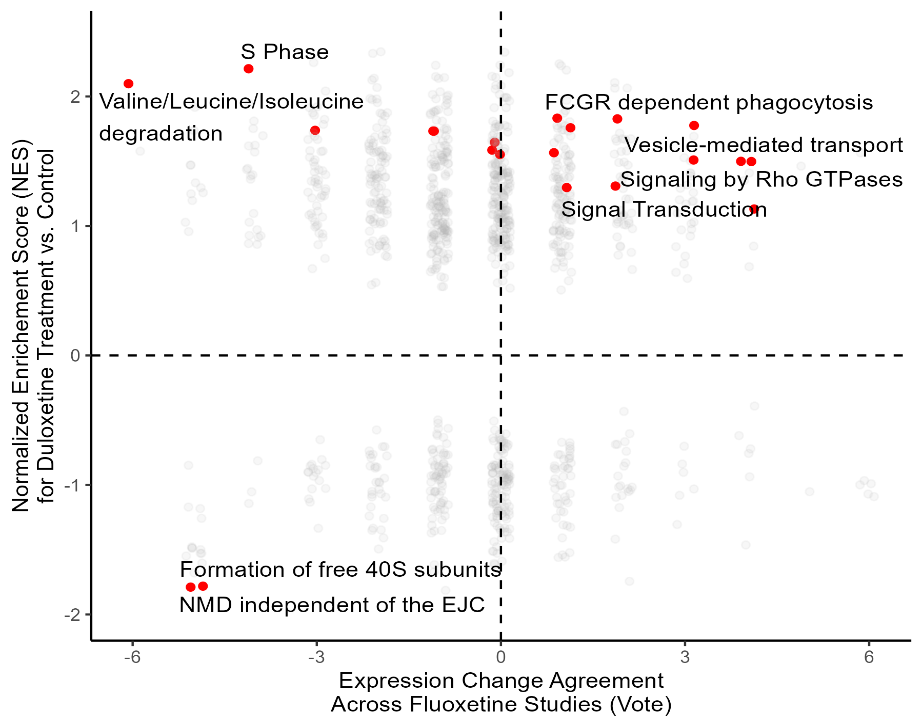


**A.**

**B.**


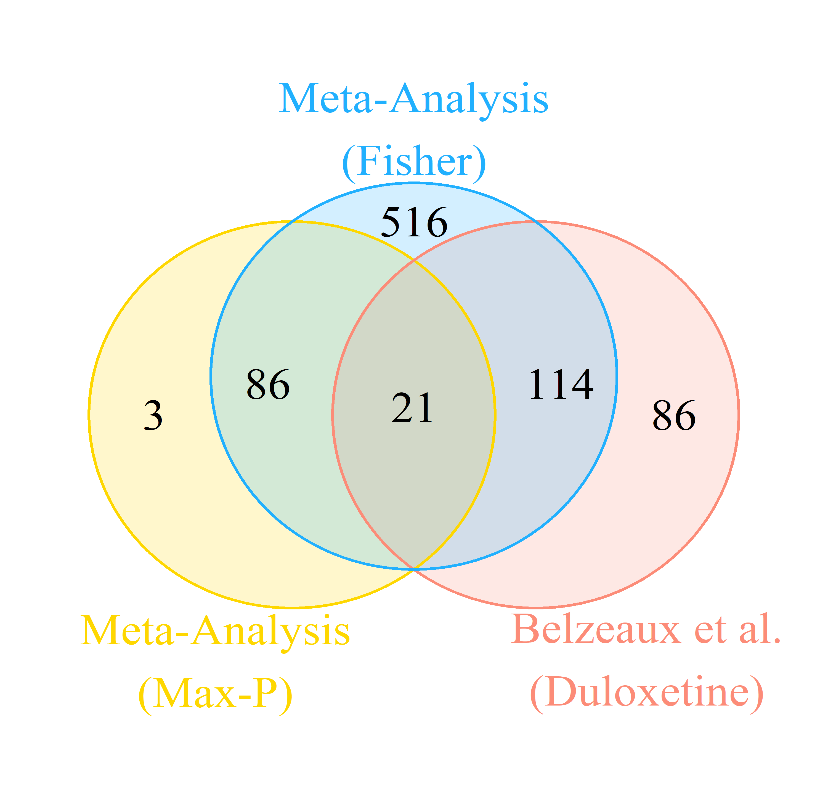


Supplementary Figure 11. (A) Overlap of pathways identified as statistically significant in comparison of duloxetine- vs. placebo-treated MDD patients (q < 0.05), with pathways identified by Fisher’s and Max-P meta-analysis of stressed/depressed subjects treated with fluoxetine vs. control. (B) Scatterplot comparing Normalized Enrichment Score (NES) of duloxetine- vs. placebo- treated subjects against expression change agreement across fluoxetine vs. control studies included in meta-analysis. Positive Vote or NES indicates upregulation with treatment. 21 pathways identified as statistically significant by Max-P and within duloxetine study (q<0.05) are highlighted in red.


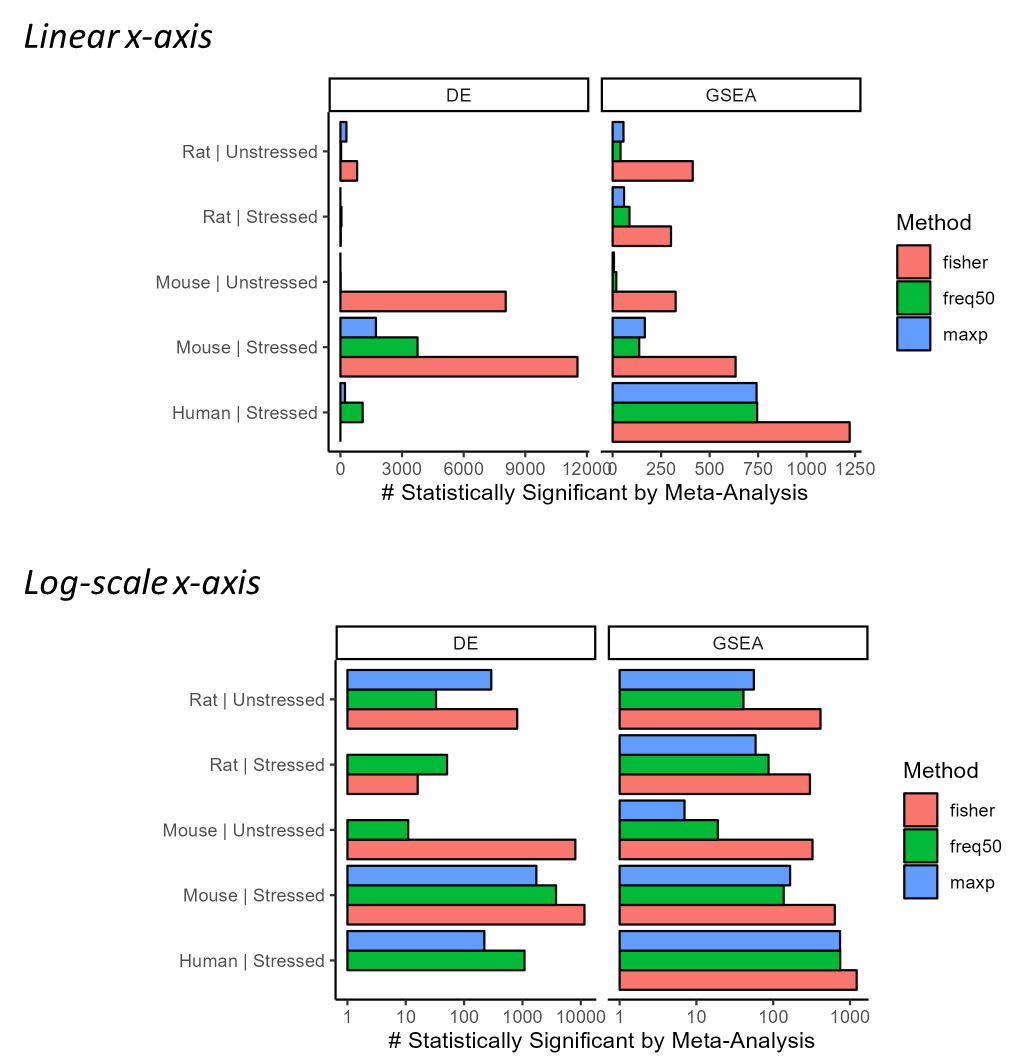


Supplementary Figure 12. Number of genes (“DE”) or pathways (“GSEA”) identified as statistically significant in each meta-analysis, with q < 0.05. Same data are presented in linear (*top*) and log10 scale (*bottom*).

**A.**


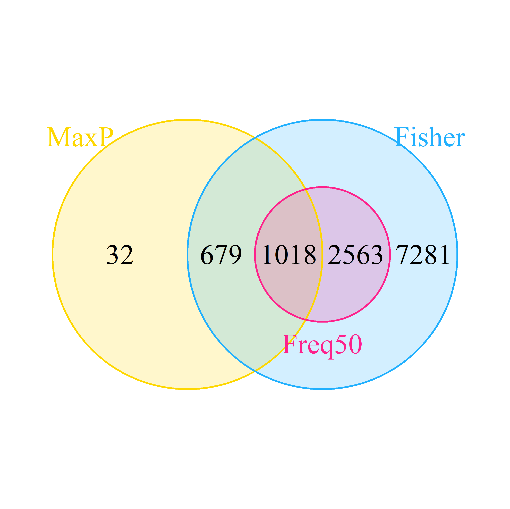

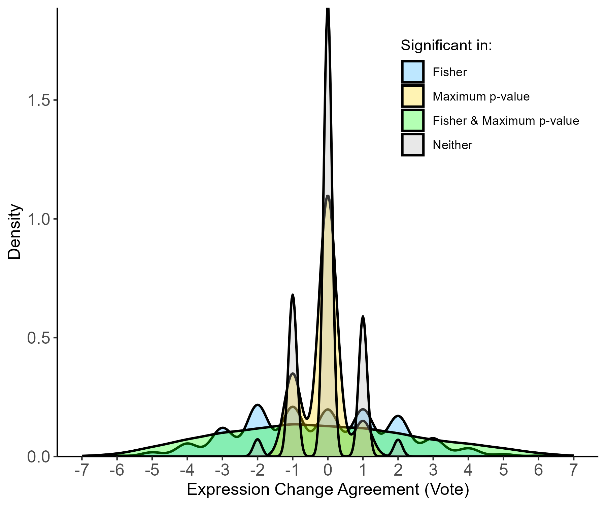


**B.**


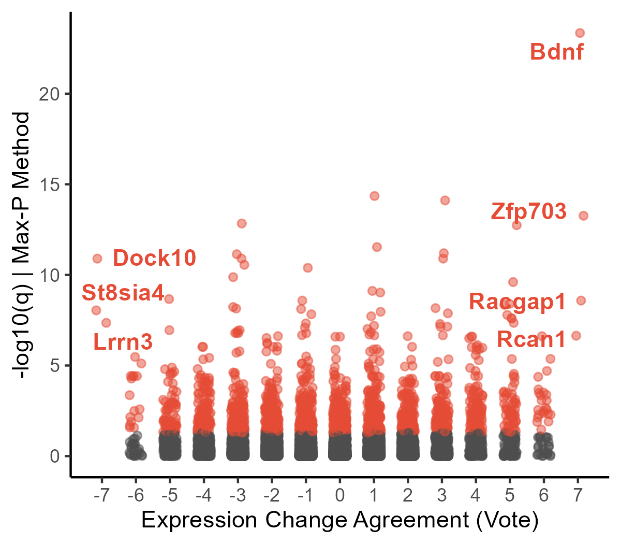


**C.**

Supplementary Figure 13. (A) Genes identified as differentially expressed (q < 0.05) across the seven comparisons of treatment effects in stressed mice. (B) Density plot showing distribution of Vote Sums across genes, colored by meta-analysis result. (C) Volcano plot of meta-analysis results for differential expression of fluoxetine vs. control in stressed mice. Genes with absolute vote sum of 7 are labeled.


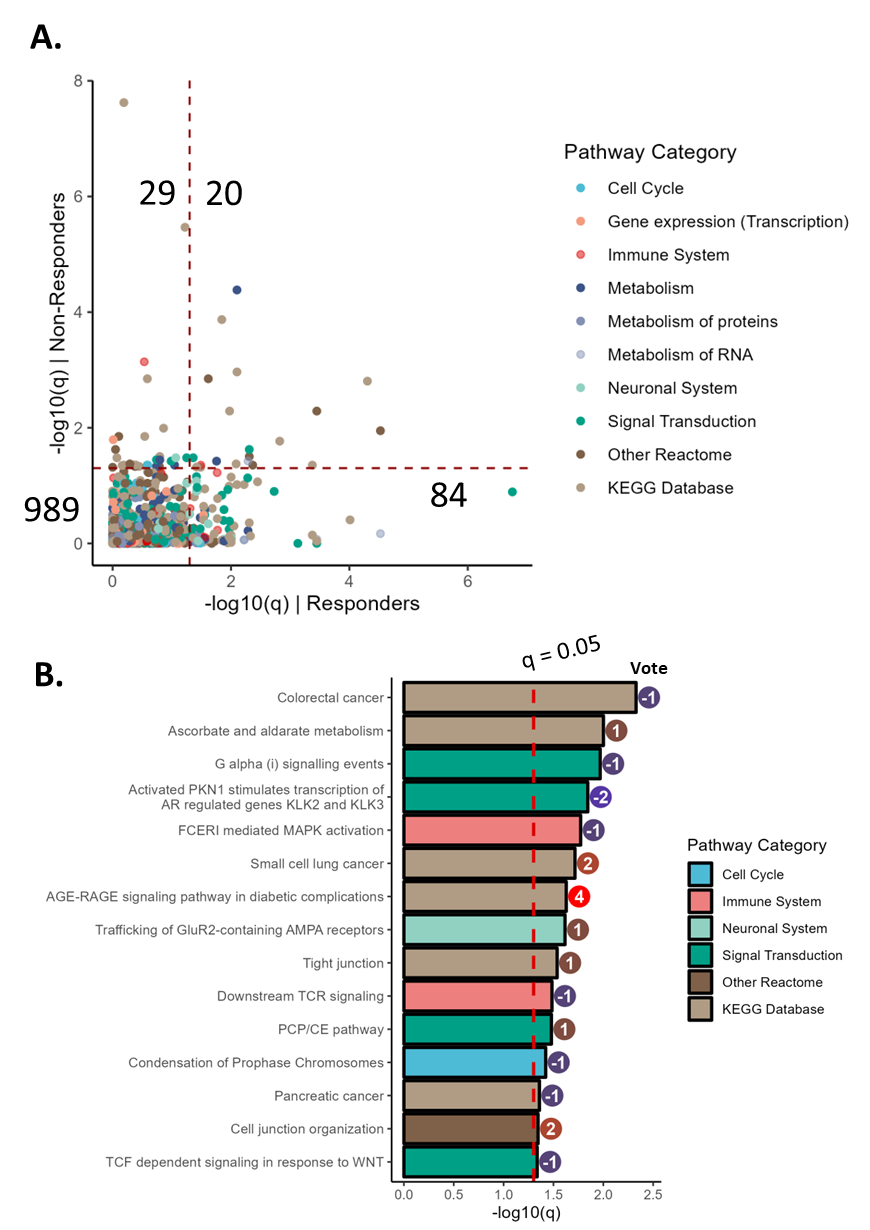


Supplementary Figure 14. (A) Comparison of pathway enrichment by treatment for responders and non-responders, by Max-P. Dotted lines indicate q = 0.05, and counts of pathways within each quadrant are provided. (B) q-value by Max-P and Vote Sums for pathways identified as significant across the eight comparisons of treatment vs. control in responders (q < 0.05 by Max-P), but consistently *not* significant across eight comparisons of treatment vs. control for non-responders (q > 0.05 by both Max-P and Fisher’s method).


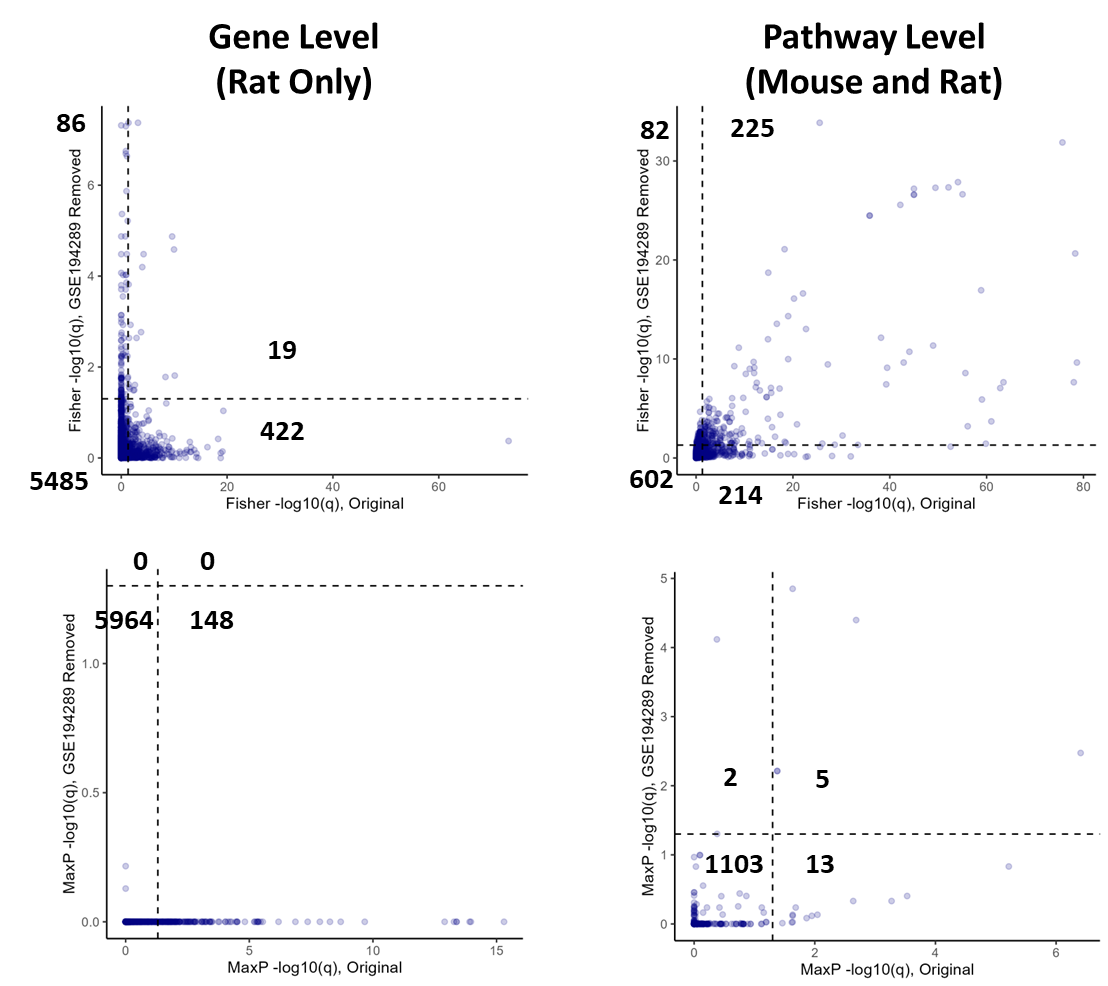


Supplementary Figure 15. Sensitivity analysis removing DS19 (profiling of 27 brain regions by Rayan *et al.*). Gene (*left*) and pathway-level (*right*) analyses are presented. Dashed lines indicate q=0.05, diagonal line indicates y=x. Number in each quadrant indicates number of genes or pathways in that quadrant. Results of Fisher’s meta-analysis (*top*) and Max-P (*bottom*).


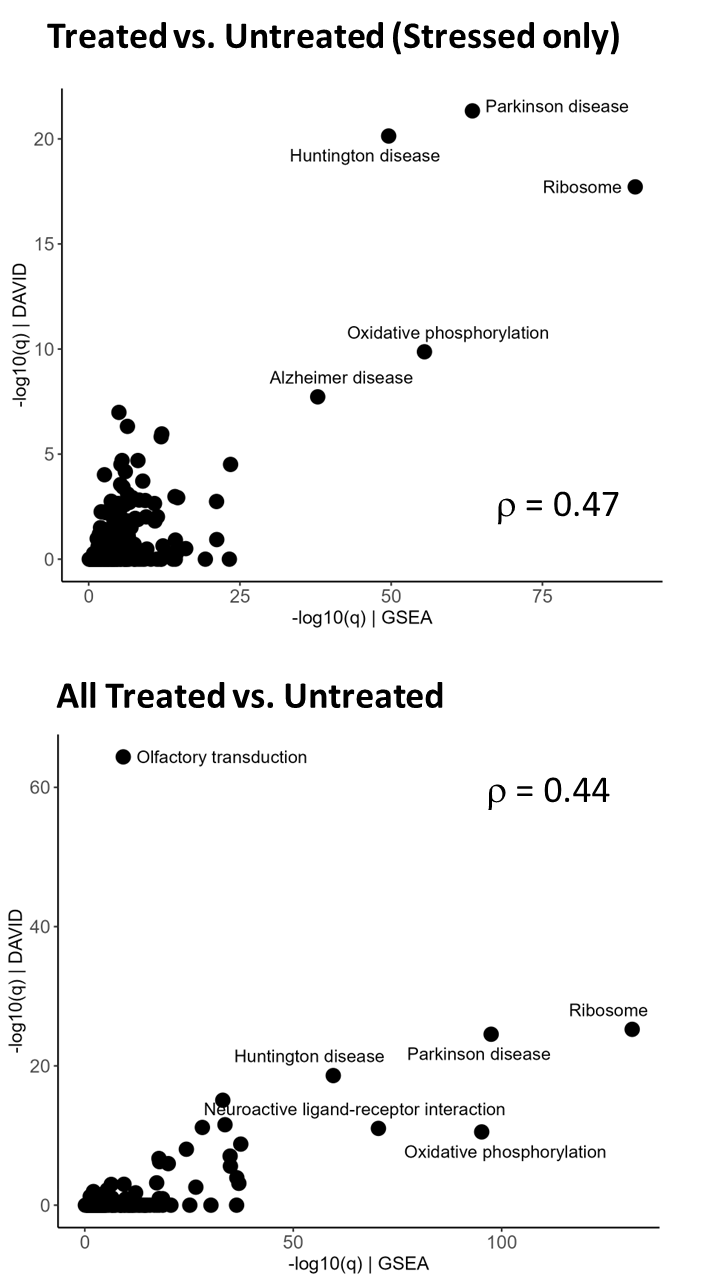


Supplementary Figure 16. Comparison of q-values from Fisher’s meta-analyses of GSEA and DAVID functional enrichment tool results for all treated vs. untreated (*top*) and stressed only (*bottom*).
